# Eppur Si Muove: Evidence for an External Granular Layer and Possibly Transit Amplification in the Teleostean Cerebellum

**DOI:** 10.3389/fnana.2016.00049

**Published:** 2016-05-02

**Authors:** Daniela Biechl, Alessandro Dorigo, Reinhard W. Köster, Benedikt Grothe, Mario F. Wullimann

**Affiliations:** ^1^Division of Neurobiology, Department Biology II, Ludwig-Maximilians-Universität MünchenMunich, Germany; ^2^Institute of Zoology, Cellular and Molecular Neurobiology, Technische Universität BraunschweigBraunschweig, Germany

**Keywords:** calcium binding proteins, cerebellum, eurydendroid cells, Purkinje cells, ptf1a, phospho-histone H3, zebrafish, Zebrin II

## Abstract

The secreted signaling factor Sonic Hedgehog (Shh) acts in the floor plate of the developing vertebrate CNS to promote motoneuron development. In addition, *shh* has dorsal expression domains in the amniote alar plate (i.e., in isocortex, superior colliculus, and cerebellum). For example, *shh* expressing Purkinje cells act in transit amplification of external granular layer (EGL) cells of the developing cerebellum. Our previous studies had indicated the presence of an EGL in anamniote zebrafish, but a possible role of *shh* in the zebrafish cerebellar plate remained elusive. Therefore, we used an existing zebrafish transgenic line Tg(*2.4shha-ABC-GFP*)sb15; Shkumatava et al., 2004) to show this gene activity and its cellular localization in the larval zebrafish brain. Clearly, GFP expressing cells occur in larval alar zebrafish brain domains, i.e., optic tectum and cerebellum. Analysis of critical cerebellar cell markers on this transgenic background and a PH3 assay for mitotic cells reveals that Purkinje cells and eurydendroid cells are completely non-overlapping postmitotic cell populations. Furthermore, *shh-*GFP cells never express Zebrin II or parvalbumin, nor calretinin. They are thus neither Purkinje cells nor calretinin positive migrating rhombic lip derived cells. The *shh-*GFP cells also never correspond to PH3 positive cells of the ventral cerebellar proliferative zone or the upper rhombic lip-derived EGL. From this marker analysis and the location of *shh-*GFP cells sandwiched between calretinin positive rhombic lip derived cells and parvalbumin positive Purkinje cells, we conclude that *shh-*GFP expressing cells qualify as previously reported *olig2* positive eurydendroid cells, which are homologous to the amniote deep cerebellar nuclei. We confirm this using double transgenic progeny of *shh*-GFP and *olig2*-dsRed zebrafish. Thus, these zebrafish eurydendroid cells may have the same role in transit amplification as Purkinje cells do in amniotes.

## Introduction

During amniote cerebellar development, early Purkinje cells have been reported to secrete Sonic Hedgehog (Shh) via their dendritic tree which signals to external granular layer (EGL) cells to maintain proliferation (transit amplification; Dahmane and Ruiz i Altaba, 1999; Dahmane et al., 2001; Ruiz i Altaba et al., 2002; Kriegstein and Alvarez-Buylla, 2009). Although in teleosts, a superficial EGL in the larval cerebellar plate has been reported in addition to a ventral cerebellar proliferation zone (Mueller and Wullimann, 2002, 2003, 2005; Wullimann and Mueller, 2002; Wullimann et al., 2011), a similar role of *shh* remains elusive in the anamniote cerebellum, because *shh* expression has neither been reported or characterized in this brain part. For the present paper, we used an existing transgenic *shh*-GFP zebrafish line (Shkumatava et al., 2004) and analyzed many cerebellar cell type markers together with the highly specific mitotic cell marker phospho-histone H3 (PH3) on this transgenic background in order to confirm and clarify (1) the location of cerebellar mitotic cells (and hence proliferation zones) and of differentiated cerebellar cell types, (2) the existence of early cerebellar *shh* expressing cells and (3) the eventual identity of cerebellar *shh-*GFP cells.

The cerebellum is an evolutionary novelty for gnathostome vertebrates because agnathans lack this brain structure (Nieuwenhuys and Nicholson, 1998; Wicht and Nieuwenhuys, 1998). The characteristic cerebellar **three-layered histology** in teleosts is highly reminiscent of the amniote situation. An inner granular, an intermediate Purkinje cell and a superficial molecular layer (Meek, 1992; Meek and Nieuwenhuys, 1998) are clearly present in the teleostean corpus cerebelli. In the zebrafish cerebellum, Purkinje cells form a continuous sheet from the (caudal) vestibulolateral lobe through corpus and into the (anterior) valvula cerebelli. This sheet at the interface of molecular and granular layer is folded into a mirror imaged S-shape (Miyamura and Nakayasu, 2001). However, in the vestibulolateral lobe and valvula cerebelli of other teleost species, Purkinje cells may be dislocated toward the molecular layer (Pouwels, 1978; Meek, 1992). The valvula cerebelli is a phylogenetically derived cerebellar subdivision for ray-finned fishes. Also cartilaginous fish have these three cerebellar histological compartments (molecular, Purkinje, granular layer), but their granular layer mostly aggregates in the medial cerebellum (Fiebig, 1988; Smeets, 1998; Rodríguez-Moldes et al., 2008).

The teleostean three-layered cerebellar cortex results from its characteristic cytoarchitectonics. The deeply lying inner granule cells emit a parallel fiber that ascends to and bifurcates in the superficial molecular layer. Inhibitory Golgi interneurons are interspersed among the granule cell bodies. The inhibitory Purkinje cells at the interface of these two layers extend a large dendritic tree into the molecular layer, which runs perpendicular to the bifurcating parallel fibers and the molecular layer contains inhibitory stellate cells. Different from amniotes, teleosts lack excitatory deep cerebellar output nuclei. Large excitatory neurons with this efferent role, so-called eurydendroid cells, are present within or slightly below the teleostean Purkinje cell layer (which is therefore alternatively called ganglionic cell layer). A further cytoarchitectonic difference to amniotes is that inhibitory basket cells in the lower molecular layer are absent in teleosts (Villani et al., 1982; Meek and Nieuwenhuys, 1991; Ikenaga et al., 2005; Meek et al., 2008).

Regarding **transmitters and neurochemistry**, teleostean Purkinje cells, Golgi and stellate cells are GABAergic, whereas granule cells are glutamatergic and eurydendroid cells glutamatergic or aspartatergic (Anzelius et al., 1995: GABA_A_ receptor ß2/ß3 subunits, Mikami et al., 2004: glutamate receptor δ2, and *GluRδ2*; Martyniuk et al., 2007: *GAD 65/67*; Ikenaga et al., 2005: GABA, aspartate; Meek et al., 2008: glutamate, GABA, GAD, GABA transporter 1, metabotropic glutamate receptors1α and 2/3; Delgado and Schmachtenberg, 2008: GABA, GAD65, GABA_Aα1_, and GABA_B1_ subunits; Bae et al., 2009: *gad1/2*, *vglut2a*). Further, neurotoxic effects of kainic acid only affect inhibitory Purkinje and Golgi cells, but not granule and eurydendroid cells (the “insensitive Purkinje cells” of Villani et al., 1982). Moreover, retrograde backlabel from cerebellar projection target areas reveals goldfish eurydendroid cells as the efferent cerebellar cells, which in double-label assays are never immunopositive for GABA or for aldolase C (Zebrin II, a specific Purkinje cell marker; Ahn et al., 1994), but about half of them are aspartate (Ikenaga et al., 2005, 2006) positive, whereas others are glutamate immunopositive (Meek et al., 2008). There are no cerebellar glycinergic neurons in teleosts, because there are neither cerebellar transgenically GFP-labeled glycine transporter 2 cells (Barreiro-Iglesias et al., 2013) nor is any cerebellar glycin receptor expression observed (Imboden et al., 2001). Additionally, the calcium-binding protein parvalbumin is selectively found in teleostean Purkinje cells, while calbindin characterizes granular cells in the cerebellar caudal lobe (Alonso et al., 1992; Porteros et al., 1998; Crespo et al., 1999; Bae et al., 2009). In contrast, calretinin stains a minor fraction of eurydendroid cells (Díaz-Requeira and Anadón, 2000; Castro et al., 2006; Meek et al., 2008) and many more migrating upper rhombic lip cells on their way to the isthmic medulla oblongata to form there the secondary gustatory nucleus (Volkmann et al., 2010). As mentioned above, Zebrin II reliably stains teleostean Purkinje cells (Lannoo et al., 1991a,b; Meek et al., 1992) including their axons. These reveal fine punctate terminals around intracerebellar eurydendroid cell somata plus a few axons originating from caudal Purkinje cells which form the cerebello-octavolateralis tract and exit the cerebellum to enter octavolateral nuclei (Lannoo et al., 1991a; Ikenaga et al., 2006). The eurydendroid cells are thus the excitatory cerebellar projection cells relaying Purkinje cell input to the extracerebellar brain and are interpreted as the homolog of the amniote deep cerebellar nuclei. Thus, cerebellar internal **microcircuitry** of teleostean granule cells, Golgi cells, stellate cells and Purkinje cells in teleosts is principally similar to amniotes, except for the intracerebellar location of efferent eurydendroid cells and the absence of basket cells (Mikami et al., 2004; Ikenaga et al., 2005; Han et al., 2006; Meek et al., 2008). Furthermore, eurydendroid cells have an extensive dendritic tree into the molecular layer, which is absent in amniote deep cerebellar nuclear cells.

Cerebellar **afferent connections** in teleosts (for example mossy fiber input from precerebellar, pretectal, accessory optic, and some primary sensory octavolateral nuclei as well as from the spinal cord) and **efferent connections** (to ventral thalamus, pretectum, nucleus ruber, nucleus oculomotorius) are highly comparable to amniotes (Finger, 1978a,b; Murakami and Morita, 1987; Wullimann and Northcutt, 1988, 1989; Torres et al., 1992; Ikenaga et al., 2002; Ito et al., 2003; Yang et al., 2004; Folgueira et al., 2006; Xue et al., 2007, 2008; Matsui et al., 2014). In addition, a large nucleus in the dorsal tegmentum (n. lateralis valvulae) forms a major input structure unique to the teleostean cerebellum (Yang et al., 2004). However, a pons and, thus, pontocerebellar input, is absent in teleosts, but different telencephalo-cerebellar relays have evolved independently in various teleost clades (Wullimann and Meyer, 1993; Imura et al., 2003). Further, climbing fibers arising from the inferior olive do exist in teleosts, but their terminals are somewhat restricted to Purkinje cell somata and their proximal dendrite (Castejon and Sims, 2000; Meek et al., 2008; Xue et al., 2008). Thus, distal dendrites of both Purkinje cells and eurydendroid cells in the molecular layer receive massive parallel fiber input, but no climbing fiber input (Meek et al., 2008).

Turning now to **cerebellar development**, the vertebrate rhombic lip comes into play. It is a unique embryonic proliferative zone of the dorsalmost alar plate hindbrain bordering the early rhombic groove. The amniote rhombic lip gives rise to various cell masses that partially undergo long tangential migrations before settling in their remote adult locations (see review by Wullimann et al., 2011). The so-called lower rhombic lip lines the rhombic groove bilaterally and fuses anteriorly to form a transverse ridge, the upper rhombic lip, which develops mostly into the cerebellum (see Section “Discussion”). The lower rhombic lip is longitudinally and dorsoventrally regionalized into various generative zones, which differ in transcription factor gene expression. Generally, a dorsal rhombic lip zone expressing *math1* (*atoh1*) is complemented by a ventral one expressing *ptf1a*. In the mammalian anterior part of the lower rhombic lip these two zones give rise to the excitatory and inhibitory cochlear nuclear cells, respectively. In the posterior part of the lower rhombic lip, the *math1* expressing zone generates precerebellar (for example the pontine) cell masses, whereas the *ptf1a* expressing zone generates the inferior olive (see above).

Similarly, the amniote upper rhombic lip very early differentiates into a ventral cerebellar *ptf1a* expressing proliferation zone which forms all inhibitory cerebellar cell types (including the inhibitory fraction of deep cerebellar nuclear cells) whereas the remaining proliferative upper rhombic lip gives rise to an EGL and an early ventrally migrating population destined to form the excitatory deep cerebellar nuclear cells. All those dorsal cell masses are *math1* (*atoh1*) positive and are sometimes referred to as upper rhombic lip (excluding the cerebellar *ptf1a* zone, see discussion in Wullimann et al., 2011). Even predating the establishment of the EGL such *math1* expressing upper rhombic lip cells migrate anteroventrally in order to populate the tegmental zone of the first rhombomere and to form cholinergic cell groups, i.e., the parabigeminal nucleus, the pedunculo-pontine/laterodorsal-tegmental groups and the cholinergic cells of the parabrachial nucleus (Machold and Fishell, 2005; Wang et al., 2005; Nichols and Bruce, 2006).

Regarding **anamniotes**, recent developmental studies in the zebrafish have revealed that similar dorsal *atoh1* and ventral *ptf1*a expressing progenitor zones do exist in the upper rhombic lip (Volkmann et al., 2008; Kani et al., 2010; Hashimoto and Hibi, 2012; Hibi and Shimizu, 2012; Kaslin et al., 2013). However, a distinct qualitative difference to the amniote situation is that the teleostean efferent eurydendroid neurons originate overwhelmingly from *ptf1a* expressing progenitors and only a small fraction from *atoh1* expressing ones (Kani et al., 2010; Hibi and Shimizu, 2012). This correlates with the truncated migratory behavior of these cells, since they remain within the cerebellum instead of migrating below it as in amniotes (see above). In a previous paper, we have studied the dynamics of *wnt1*/*atoh1a* expressing zebrafish upper rhombic lip derived cell migration and found that such very early migrating cells are cholinergic and show connections known for the adult nucleus isthmi, secondary gustatory nucleus and superior reticular nucleus (Volkmann et al., 2010), the homologs of the mammalian parabigeminal nucleus, parabrachial nucleus, and the pedunculo-pontine/laterodorsal-tegmental groups (Mueller et al., 2004). Somewhat later, upper rhombic lip/EGL cells at the subpial periphery of the cerebellar plate which express *atoh1* genes continue extendedly to migrate ventrally to populate the internal granule cell layer (Köster and Fraser, 2001, 2004, 2006; Rieger et al., 2008; Kani et al., 2010). In contrast, *ptfa1* expressing cells in the ventral cerebellar proliferation zone are fated to become Purkinje and other inhibitory cerebellar cells (Kani et al., 2010). Overall, this is an impressively similar spatiotemporal sequence of upper rhombic lip differentiation events when compared to amniotes (see review in Wullimann et al., 2011).

However, it has been questioned whether or not there is a true **EGL** in ray-finned fishes (Chaplin et al., 2010; Butts et al., 2014). In amniotes, early Purkinje cells have been reported to secrete Shh via their dendritic tree signaling to EGL cells to maintain proliferation (transit amplification; Dahmane and Ruiz i Altaba, 1999; Dahmane et al., 2001; Ruiz i Altaba et al., 2002; Kriegstein and Alvarez-Buylla, 2009; see Section “Discussion”). Various papers reported on the existence of a ventral cerebellar proliferation zone and a superficial EGL in the larval cerebellar plate (Mueller and Wullimann, 2002, 2003, 2005) and while support by gene expression studies followed suit (Wullimann and Mueller, 2002; Wullimann et al., 2011; see Section “Discussion” there), the expression of *shh* in the early cerebellum during rhombic lip proliferation remained elusive. Hence, for the present paper, we availed ourselves of existing transgenic *shh*-GFP zebrafish line (Shkumatava et al., 2004) and studied larval cerebellar development using various specific cerebellar cell type markers and the highly specific mitotic cell marker phospho-histone H3 (PH3) on this transgenic background in order to confirm and clarify (1) the location of cerebellar mitotic cells, and hence proliferation zones, and how they relate to differentiated cerebellar cell types, (2) the existence of early alar plate (e.g., cerebellar) *shh* expressing cells and (3) the eventual identity of cerebellar *shh-*GFP cells. To this end, we furthermore used progeny of transgenic *shh*-GFP fish mated with transgenic *olig2*-dsRed fish (see: “Transgenic Zebrafish Strains” in Section “Materials and Methods”). We found that a cerebellar ventral proliferation zone in addition to an EGL is confirmed with phospho-histone H3 and that the cerebellar plate *shh-*GFP cells correspond to the *olig2* expressing cerebellar eurydendroid cells.

## Materials and Methods

### Animal Maintenance

Wild-type zebrafish were kept in a zebrafish housing system (ZebTEC, stand-alone-unit, Tecniplast©) at a temperature of 28°C and a 12/12 light/dark cycle at the Ludwig Maximilians University Munich (LMU). Animals used in this study were treated according to the German regulations on Animal Protection (Deutsches Tierschutzgesetz). Experiments conducted in this paper involved no animal experiments in the sense of the German Animal Protection law. We used dead animals and fixed brain tissue to conduct the immunohistochemical procedures. Transgenic fish lines used (see below) were generated previously in other laboratories. Maintenance and breeding of transgenic fish were approved by local government authorities. All experiments involving zebrafish embryos and larvae were conducted in accordance with legal regulations (EU Directive 2010_63).

### Transgenic Zebrafish Strains

The transgenic line Tg(2.4*shh*a-*ABC-GFP*)sb15 was originally published as Tg(*2.2shh:gfpABC#15*) by Shkumatava et al. (2004; see there for details on its generation) and will be referred to in the following as *shh-*GFP line. Transgenic specimens were bred and raised by Dr. Thomas Mueller (Albert Ludwigs-University, Freiburg i. Br., Germany) up to 2, 3, 4, and 5 days post fertilization (dpf) and then fixed in 4% paraformaldehyde in PB (see below). Heterozygous carriers of the Tg(*olig2:dsred2*)vu19 (Kucenas et al., 2008) were bred to heterozygous Tg(2.4*shh*a-*ABC-GFP*)sb15 carriers and double transgenic embryos were sorted according to their green and red fluorescence (at TU Braunschweig, Germany). After 12 h the medium was replaced with 0.003% PTU (*N*-Phenylthiourea, Sigma–Aldrich) in 30% Danieau and changed daily. The former line will be referred to as *olig2*-dsRed in the following. Similarly, heterozygous carriers of Tg(*PC:FyntagRFP-T*) zebrafish (see Matsui et al., 2014) were crossed with corresponding Tg(*olig2:EGFP*)vu12 (Shin et al., 2003) zebrafish and their double-transgenic offspring selected accordingly (as described above). The former line will be referred to as *PC*-tagRFP below. The line visualizes the cell membrane of Purkinje cells, because a regulatory element derived from the zebrafish carbonic anhydrase 8 gene, specifically expressed in Purkinje cells, is involved (Matsui et al., 2014; see also Bae et al., 2009). Histological processing of certain transgenic specimens took place at the LMU. It should be noted that transgenic larvae were kept as far as possible in darkness during histological processing due to the photosensitive GFP-protein.

### Animal Preparation and Cutting Procedure

Adult wild-type zebrafish (4) were sacrified by decapitation after anesthesia with an overdose of MS-222 (ethyl 3-aminobenzoate, Sigma–Aldrich). Brains were exposed dorsally by removing the skull and fixed with freshly prepared cold 4 % paraformaldehyde (PFA) in Sörensen phosphate buffer (PB, pH 7.4) at 4°C overnight before being removed from the skull.

Zebrafish larvae (98) wild-type and transgenic animals; see above) were killed with an overdose of tricaine methanesulfonate (MS-222) and fixed in paraformaldehyde (4% PFA in PB) at 4°C overnight. Following cryoprotection in sucrose solution (30% sucrose solution at 4°C overnight, adult brains and larvae embedded in TissueTek (tissue freezing medium, A. Hartenstein GmbH) and cryosectioned (Leica, CM 3050 S) at 14 μm (larvae) or 30 μm (adult) before thaw mounted onto Superfrost Plus glass slides (Thermo) and coverslipped.

### Immunohistochemical Processing

Immunohistochemical incubations were done in a humid chamber. After washing off TissueTek in cryosections in phosphate buffered saline (PBS), endogenous peroxidase activity was first blocked with 0.3% H_2_O_2_ in PBS for 30 min at room temperature (RT), washed in PBT (PBS + 0.1% Tween 20) and blocked with blocking buffer (2% normal goat serum, 2% bovine serum albumin, 0.2% Tween 20, 0.2% Triton X-100 in PBS) for 1 h at RT before exposition to a primary antibody diluted in blocking buffer at 4°C for 1–3 days (dilution see **Table 1**). After washing in PBT, the sections were incubated with the secondary antibody (see **Table 2**) diluted in blocking solution overnight at 4°C. In case of double- or triple- immunofluorescence, a second and third primary antibody was sequentially applied after intermittent washing in PBT and blocking. Subsequently, one or two corresponding secondary antibodies diluted in blocking buffer were applied overnight, after intermittent washing in PBT and blocking (see above for details). Finally, sections were washed in PBT and counterstained with DAPI (40-6-diamidino-2-phenylindole; Carl Roth, 1:1000) or in case of triple immunostains with TO-PRO (Molecular probes, 1:1000; not shown) and washed in PBS. A few stains were performed with Neurotrace (a fluorescent Nissl stain; Molecular Probes; 1:100) as an additional counterstain to DAPI. Slides were then mounted with Vectashield (Vectorlabs) and coverslipped. Regarding antibodies against calcium-binding proteins (parvalbumin, calretinin), we have previously performed various controls and Western blot analysis (see Kress et al., 2014) and the same is true for the antibody against tyrosine hydroxylase (Yamamoto et al., 2010, 2011). We furthermore checked for differences between the intrinsic GFP signal with the one enhanced through use of the anti-GFP antibody and found no qualitative difference.

**Table 1:** Primary antibodies.

Antibody	Host	Company	Dilution
Zebrin II	Mouse, monoclonal	Gift from Richard Hawkes	1:500
Ptf1a	Goat, polyclonal	Acris, #AP16594PU-N	1:100
Parvalbumin	Mouse, monoclonal	Millipore, # 1572	1:500
Calretinin	Rabbit, polyclonal	SWANT, #7699/3H	1:500
PH3	Rabbit, polyclonal	Millipore, #06-570	1:250
GFP	Chicken	Aves Labs, #GFP-1020	1:500
Tyrosine hydroxylase	Mouse, monoclonal	AbCys, #MA318	1:500

**Table 2:** Secondary antibodies.

Antibody	Host	Company	Dilution
Anti mouse Cy3	Donkey	Dianova, #715-166-151	1:500
Anti rabbit Cy3	Donkey	Dianova, # 711-165-152	1:400
Anti goat AMCA	Donkey	Dianova, # 705-156-147	1:100
Anti chicken Alexa 488	Goat	Mol. Probes, # A11039	1:400

### Confocal Microscopy

Sectioned material: Optical sections were acquired with a Leica TCS SP-5 confocal laser-scanning microscope (Leica Microsystems, Mannheim, Germany). All microscopic images used in this study were processed to RGB stacks and projections by using ImageJ and slightly adapted for brightness and contrast with either ImageJ or Corel PHOTO-PAINT. Photographic plates were mounted and further processed into figures with CorelDRAW 9.0 (Corel Corporation).

Larvae selected for confocal microscopy analysis were anesthetized in 0.016% Tricaine (Ethyl 3-aminobenzoate methanesulfonate, Sigma–Aldrich) dissolved in 30% Danieau. Anesthetized larvae were embedded in 1.5% low melting agarose (Type IX-A, Sigma–Aldrich) in a 0.13–0.16 mm cover glass bottom Petri dish (Menzel–Gläser) and overlaid with 30% Danieau.

Imaging of zebrafish larvae (shh:EGFP/olig2:dsRed and olig2:EGFP/PC:tagRFP double-transgenic fish) was performed using an inverse Leica TCS SP8 laser scanning confocal microscope using either a 20X (0.75 NA) oil immersion objective or a 40X (1.1 NA) water immersion objective. For live imaging, 5 dpf zebrafish larvae were anesthetized in Tricaine methanesulfonate (Sigma–Aldrich) and mounted in 1.5% low melting agarose (Type IX-A, Sigma–Aldrich). To record EGFP fluorescence, the excitation wavelength was set to 488 nm and the emission bandpass filters to 495–545 nm. To record dsRed fluorescence, the excitation wavelength was set to 561 nm and the emission bandpass filters to 595 – 650 nm. Z-stacks images were collected with the confocal pinhole set to 1.0 airy unit (AU; 20x z-section of: 3.12 μm, 40x z-section of: 1.27 μm)). After imaging, larvae were fixed in 4% PFA as described above.

## Results

### *Shh-*GFP Line Visualizes Conventionally Known Ventral Central Nervous Expression Domains As Well As Dorsal Alar Plate Domains

**FIGURE 1:**
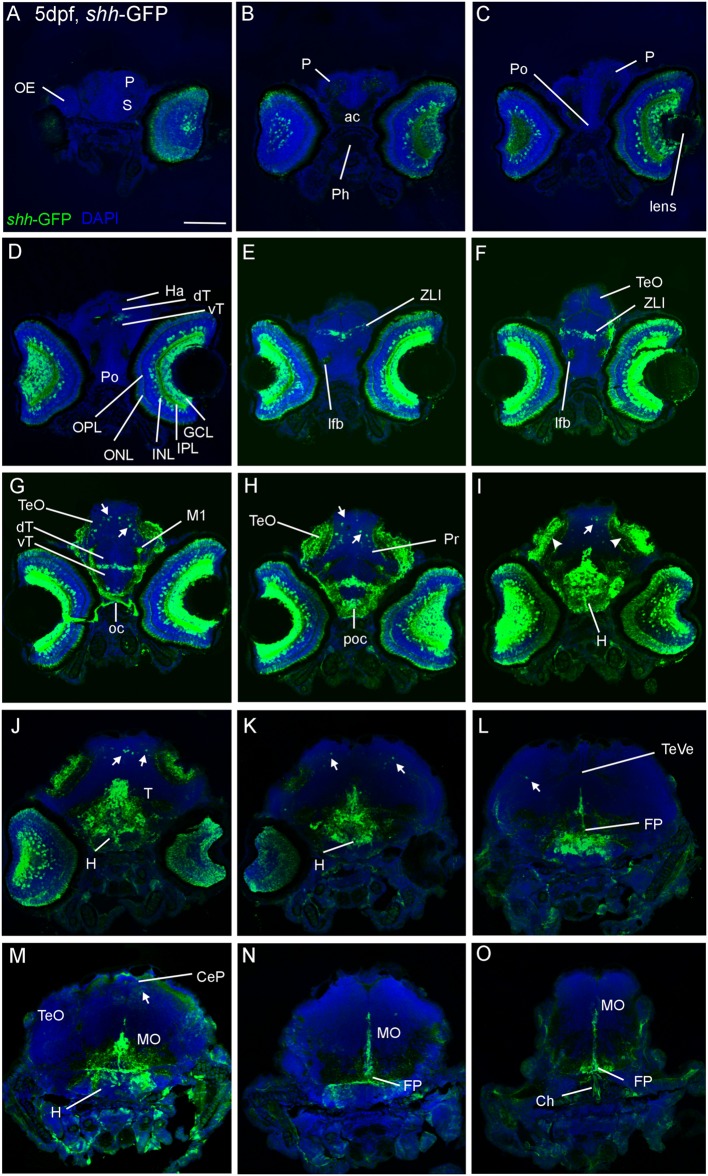
**Confocal photomicrographs (maximum projections) of transverse sections of a *shh-*GFP zebrafish larva at 5 dpf immunostained for GFP (with DAPI as counterstain) show levels from telencephalon to medulla oblongata (A–O).** Note in particular basal expression in floor plate **(J–O)**, hypothalamus **(I–M)**, and dorsal (alar plate) tectal **(G–I)** and cerebellar **(J–M)** GFP-expressing cells (arrows). Arrowheads indicate primary visual projections in optic tectum. See text for more details. Scale bar in **(A)**: 100 μm (applies to all panels). CeP, cerebellar plate; dT, dorsal thalamus; FP, floor plate; GCL, ganglion cell layer; H, hypothalamus; Ha, habenula; INL, inner nuclear layer; IPL, inner plexiform layer; MO, medulla oblongata; oc, optic chiasma; OC, otic capsule; ONL, outer nuclear layer; P, pallium; poc, postoptic commissure; PTd, dorsal part of posterior tuberculum; S, subpallium; T, midbrain tegmentum; TeO, optic tectum; TeVe, tectal ventricle; vT, ventral thalamus (prethalamus); ZLI, zona limitans intrathalamica.

**FIGURE 2:**
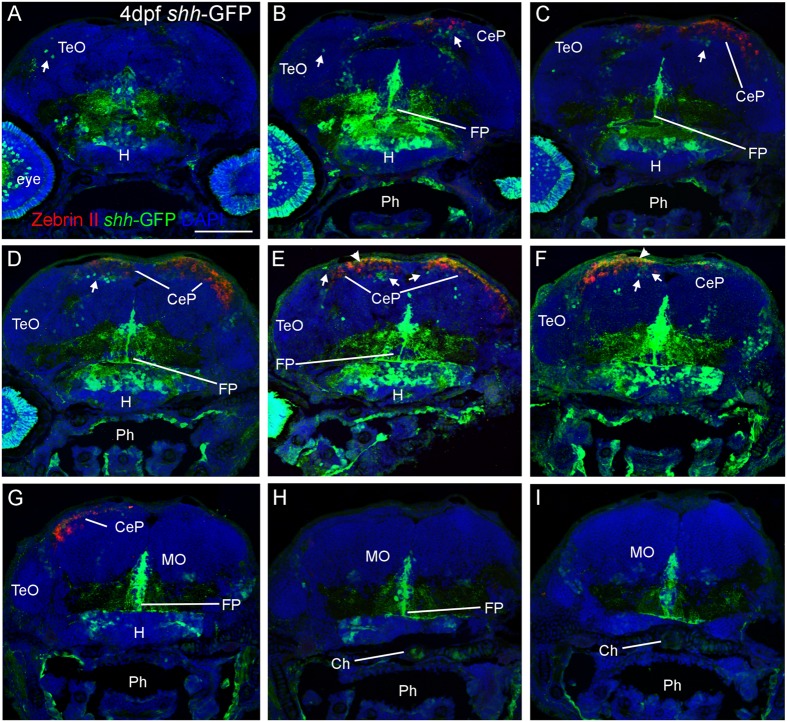
**Confocal photomicrographs (maximum projections) of transverse sections from tectal **(A)** to hindbrain **(I)** levels of a *shh-*GFP zebrafish larva at 4 dpf immunostained for GFP (with DAPI as counterstain) reveals extent of cerebellum through additional immunostain with Zebrin II.** Arrows: dorsal GFP expressing cells. Arrowheads point out GFP-expressing dendritic arborizations. Scale bar in **(A)**: 100 μm (applies to all panels). CeP, cerebellar plate; Ch, notochord; FP, floor plate; H, hypothalamus; MO, medulla oblongata; Ph, pharynx; TeO, optic tectum.

**FIGURE 3:**
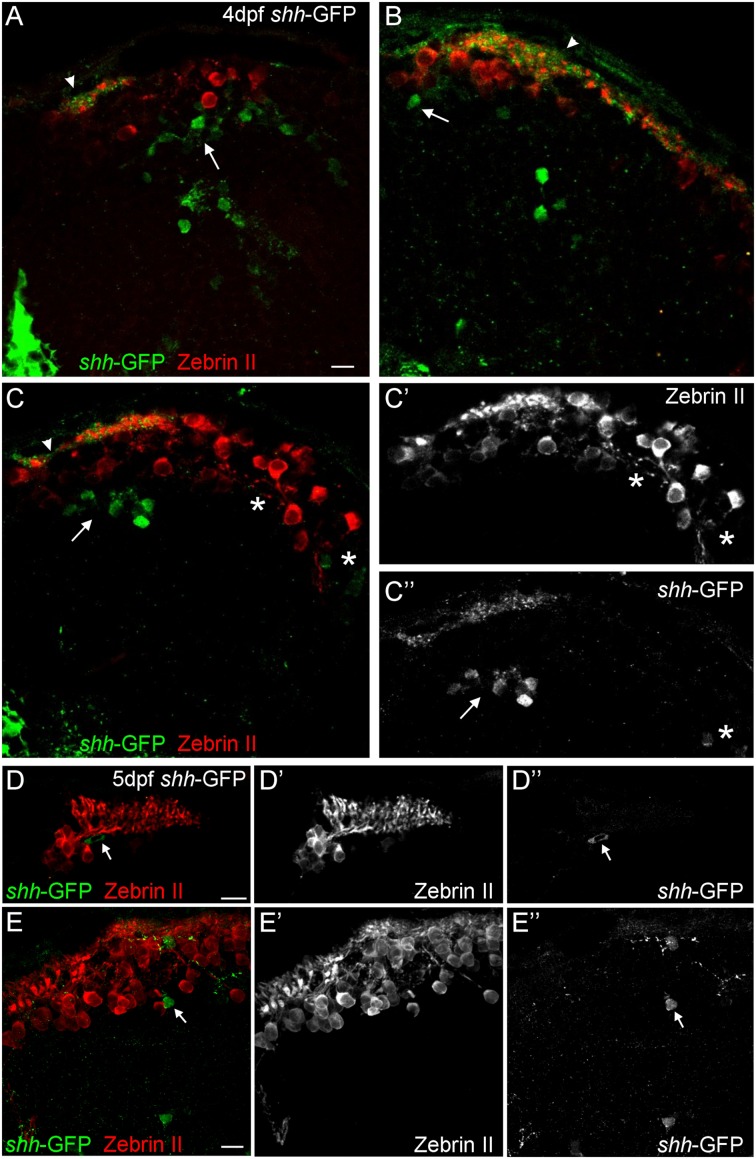
**Confocal photomicrographs (optical sections) of transverse sections of *shh-*GFP zebrafish cerebellum immunostained for Zebrin II at 4 dpf **(A–C)** and 5 dpf **(D,E)** showing that Zebrin II is not seen in *shh-*GFP expressing cells at 4 and 5 dpf.** Arrows point to GFP expressing cells. Arrowhead points out GFP-expressing dendritic arborizations. Scale bars: 10 μm, that in **(A)** also applies to **(B,C)**.

A detailed comparison of our data on the retina with those of the original description of the zebrafish *shh-*GFP line (Shkumatava et al., 2004) is given in the “Supplementary Materials” Section. Furthermore, we investigated serial cross-sections of *shh-*GFP zebrafish larvae at 2, 3, 4, and 5 dpf. At all ages, previously well established *shh* expression domains, such as the zona limitans intrathalamica, the hypothalamus, the posterior tuberculum and the floor plate extending from the tegmental midbrain into spinal cord as well as the pharynx exhibit strong GFP expression (**Figures 1** and **2**). Some more weakly GFP-stained cells are additionally seen somewhat laterally to the floor plate, in particular in the mesencephalic tegmentum (see Section “Discussion”). Interestingly, the posterior hypothalamus remains negative for a GFP signal in its ventral part. In the notochord, GFP expression is already weaker in these larval stages (and was presumably stronger in the embryonic larva). At 3 dpf, dorsal alar plate cells appear at tectal, but not yet at cerebellar levels. Also, considerable numbers of such dorsal cells are seen more caudally in the medulla oblongata (not shown). At 4 and 5 dpf, the alar GFP expressing cells are increasingly more numerous in the tectum and appear now clearly in the cerebellar plate in growing numbers (arrows in **Figures 1** and **2**). These conventionally recognized shh-expressing domains as well as the dorsal alar cells in midbrain and cerebellum show a corresponding signal in sections of wild-type zebrafish *in situ* hybridized for *shha*-mRNA (see “Supplementary Materials” Section, **Figure 2**). A fortuitous fact is that the axonal arborizations of retinal ganglion cells (which are GFP expressing, see above) are seen in the visual layers of the optic tectum (arrowheads in **Figure 1**) starting with 3 dpf and, thus, define the latter’s anteroposterior extent. GFP labeled retinal projections are also seen to reach the pretectum, in particular the developing parvocellular superficial pretectal nucleus (M1, **Figure 1G**). Another interesting detail is that GFP expressing fibers are seen in the lateral forebrain bundle (**Figure 1F**) which presumably represent ascending fibers from the posterior tuberculum (see Section “Discussion”). Within the optic tectum, the GFP expressing cell somatas are always basal to the GFP expressing retinal layers. Also, cerebellar GFP cells are in a basal position. Medullary GFP expressing dorsal cells appear to be gone at these later larval stages. Thus, our survey of transgenic *shh-*GFP zebrafish line reveals the time course and developmental profile of *shh* expressing cells via GFP fluorescence/expression along the entire anteroposterior central nervous system axis and demonstrates that dorsal alar *shh* expressing populations known from embryonic amniotes in midbrain and cerebellum do exist in addition to well established basal and floor plate domains also in larval zebrafish. Of note, *in situ* hybridized sectioned material produced in transgenic *shh*-GFP larvae (5 dpf) shows the presence of dorsal alar cerebellar GFP expressing cells which at the same time express *shha* (see “Supplementary Materials” Section, **Figure 3**).

### Zebrin II Confirms Presence of Dorsal (Alar) *shh-*GFP Cells in the Cerebellum

Next, we looked at Zebrin II, a selective marker for Purkinje cells (Brochu et al., 1990) in order to reveal the extent of cerebellar *shh-*GFP expressing cells in 4 and 5 dfp larvae (**Figure 2**). Zebrin II very reliably and effectively labels Purkinje cell bodies, dendritic trees and axons throughout the zebrafish cerebellar plate, i.e., the future corpus cerebelli. There is a considerable increase of Zebrin II expression in the 5 dpf larva compared to 4 dpf. We did neither observe Purkinje cells in the yet small larval valvula cerebelli, nor in the eminentia granularis. These data clearly reveal that *shh-*GFP cells (indicated with arrows in **Figure 2**) are present within the larval zebrafish cerebellar plate because the latter’s anteroposterior extent is delineated by Zebrin II immunopositivity. With regard to the identity of cerebellar *shh-*GFP cells, it can be clearly stated from confocal microscopical analysis that Zebrin II positive Purkinje cells are never double-labeled with GFP (see arrows in **Figure 3**), indicating that these cerebellar zebrafish *shh-*GFP cells are not Purkinje cells. Furthermore, punctate GFP staining in the most superficial molecular layer of the larval cerebellar plate (arrowheads in **Figures 2** and **3**) demonstrate that *shh-*GFP cells have processes extending into the molecular layer reminiscent of eurydendroid cells.

### Zebrin II Visualizes Cerebello-octavolateralis Tract

**FIGURE 4:**
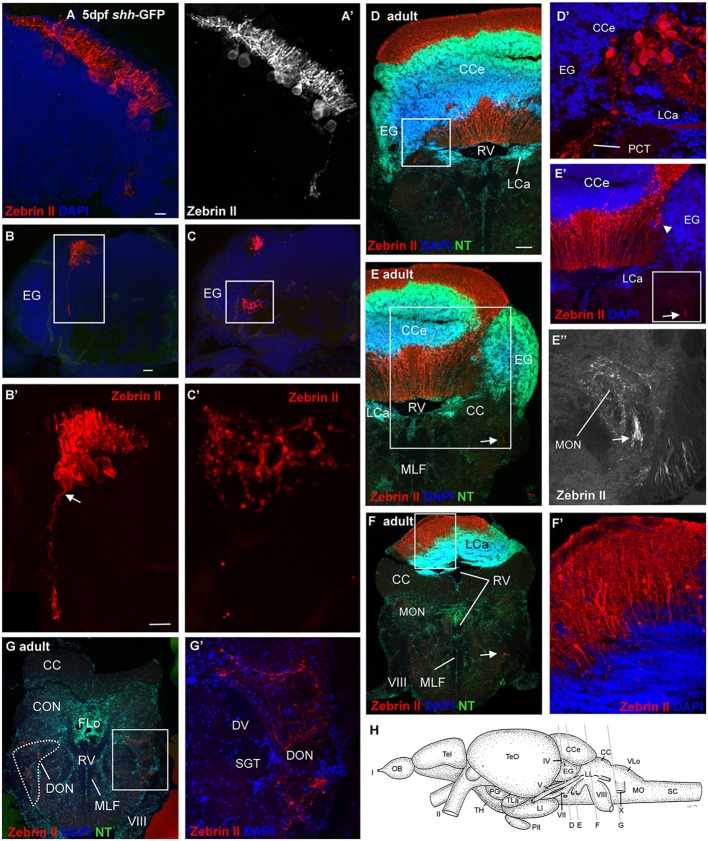
**Confocal photomicrographs (maximum projections) of transverse sections of a *shh-*GFP zebrafish cerebellum at 5 dpf **(A–C)** and sections of a wild-type adult brain immunostained for Zebrin II with DAPI and Neurotrace (NT) as counterstains **(D–G)**. (A)** Right side of larval cerebellum shows location of Purkinje cell layer. Note large cell bodies and typical dendritic trees into molecular layer. **(A’)** Monochromatic picture visualizes Purkinje cell morphology. **(B,C)** This shows more caudal cerebellar levels including eminentia granularis. **(B’)** Enlargement of caudal Purkinje cells with one exhibiting an axon directed ventrally and exiting the cerebellum. **(C’)** Enlargement of terminal field in larval vestibular primary nuclear area formed by axon shown in **(B’)**. Four transverse sections [levels indicated in drawing shown in **(H)**] from rostral **(D)** to caudal **(G)** show adult Purkinje cell bodies and dendrites in corpus cerebelli **(D,E)** and lobus caudalis cerebelli **(F,F’)**, plus some Purkinje cells along the medial edge of eminentia granularis (arrowhead in **E’**). The cerebello-octavolateralis tract is highlighted by arrows in **(E–E”,F)** and forms a terminal field in the medial octavolateralis nucleus **(E–E”)** and in the descending octaval nucleus **(G,G’)**. **(D’)** Enlargement of Purkinje cells giving rise to cerebello-octavolateralis tract (left brain side). See text for more details. Scale bars: 10 μm, that in **(B)** also applies to **(C)**, in (**D**, also applies to **E–G**): 200 μm. CC, crista cerebellaris; CCe, corpus cerebelli; DV, descending trigeminal tract; CON, caudal octavolateralis nucleus; DON, descending octaval nucleus; EG, granular eminence; FLo, facial lobe; LCa, lobus caudalis cerebelli; LI, inferior lobe; LL, lateral line nerves; MO, medulla oblongata; MON, medial octavolateralis nucleus; MLF, medial longitudinal fascicle; OB, olfactory bulb; PG, preglomerular area; Pit, pituitary; RV, rhombencephalic ventricle; SC, spinal cord; SGT, secondary gustatory tract; Tel, telencephalon; TeO, optic tectum; TH, tuberal hypothalamus; TLa, torus lateralis; VLo, vagal lobe; I, olfactory nerve; II, optic nerve; IV, trochlear nerve; V, trigeminal nerve; VII, facial nerve; VIII, octaval nerve; X, vagal nerve.

In 5 dpf zebrafish larvae, some Purkinje cells in the caudal cerebellar plate, close to the larval eminentia granularis, are observed to emit Zebrin II positive axons which project ventrally (arrow in **Figure 4B**’) and terminate with profuse arborization in the vestibular primary sensory area (**Figures 4A–C**). We went on to visualize Zebrin II positive Purkinje cells in the adult zebrafish brain (**Figures 4D–G**). Purkinje cell somata are present throughout the corpus cerebelli around the edge of the granular cells masses (**Figure 4D**). The axons of the adult cerebello-octavolateralis tract arise from a set of Purkinje cells in a position where granular masses of corpus cerebelli, lobus caudalis cerebelli and eminentia granularis meet (**Figure 4D**’) and possibly from some Purkinje cells along the medial edge of the eminentia granularis (arrowhead in **Figure 4E**’). These axons exit the cerebellum via the posterior cerebellar tract to form the cerebello-octavolateralis tract. Some Zebrin II positive Purkinje cell somata are also present at the edge of the most posterior granular masses of the lobus caudalis where they extend their dendrites into the molecular layer (**Figures 4F,F**’). The axons of the cerebello-octavolateralis tract can be followed into the medulla oblongata issuing terminals to the medial octavolateralis nucleus (**Figures 4E–E**”) and to the descending octaval nucleus (**Figures 4G,G**’), but not to the anterior, tangential, magnocellular or posterior octaval nuclei. Interestingly, within the descending octaval nucleus the terminal field is in the ventrolateral area, which is presumably vestibular and not acoustic in nature, if data from other cypriniforms are considered (Echteler, 1984, 1985; McCormick and Braford, 1994; McCormick and Hernandez, 1996; Yamamoto and Ito, 2005). Similar to the larval situation (**Figure 4C**’), the synaptic terminals appear to surround the vestibular neuronal somata in a calyx-like fashion. However, the vast majority of Purkinje cells do not issue ventrally directed axons, but tangentially oriented ones which sometimes reveal punctate terminals around unstained large cell bodies located in the Purkinje cell or upper granule cell layers, presumably representing postsynaptic eurydendroid cells (asterisks in **Figures 3C,C**’). At least in mormyrids, Purkinje cells might also synapse on other Purkinje cells (Meek et al., 1992).

### Identity of *shh-*GFP Cells

**FIGURE 5:**
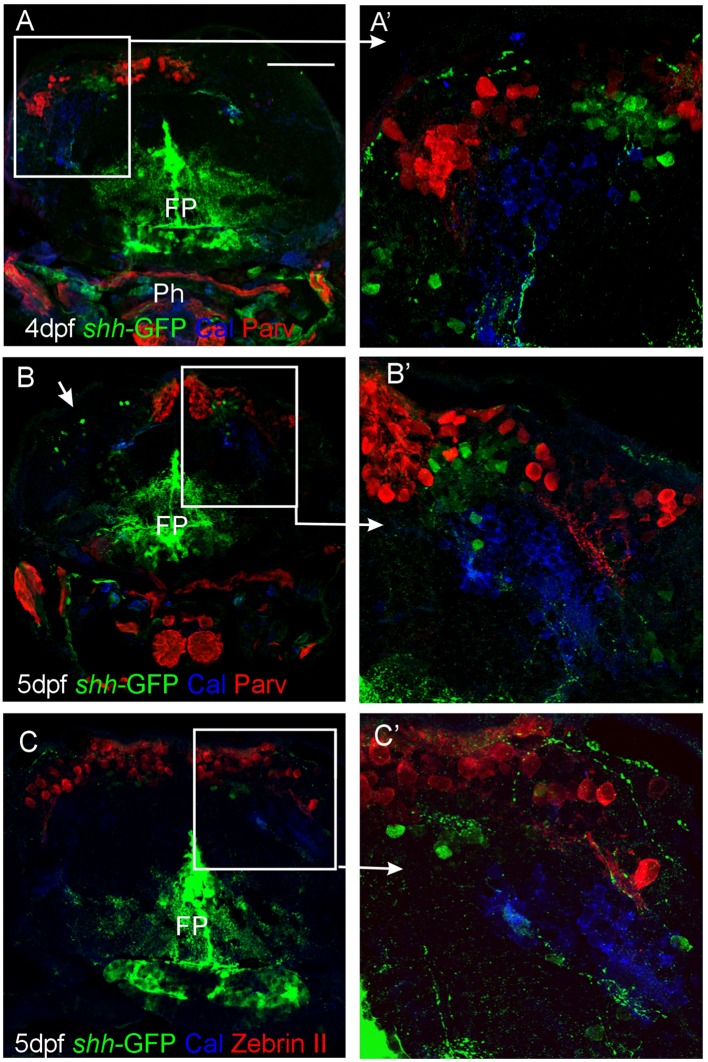
**Confocal photomicrographs (optical sections) of transverse sections of *shh-*GFP zebrafish cerebellum at 4 and 5 dpf immunostained for calretinin/parvalbumin **(A,B)** or calretinin/Zebrin II **(C)** with corresponding enlargements to show cellular details (A’–C’).** Arrows point out GFP-expressing cells. Scale bar: 100 μm (applies to **B** and **C**). FP, floor plate; Ph, pharynx.

We furthermore used immunohistochemistry for two calcium binding proteins, calretinin, and parvalbumin, to characterize the zebrafish larval cerebellar plate in *shh-*GFP transgenic larvae (**Figure 5**). These two proteins were previously established to label migrating rhombic lip cells destined for the secondary gustatory nucleus and some eurydendroid cells (calretinin) or Purkinje cells (parvalbumin), respectively (Bae et al., 2009; Volkmann et al., 2010). Our immunostains revealed clearly that neither calretinin nor parvalbumin co-localizes with cerebellar *shh-*GFP expressing cells. Furthermore, the location of calretinin positive cell bodies is ventrolateral to the more dorsally lying parvalbumin positive Purkinje cells (**Figures 5B,B**’), which is indicative of the migrating calretinin rhombic lip cells described previously (Volkmann et al., 2010). Moreover, co-stainings for Zebrin II and calretinin in *shh-*GFP larvae reveal an identical picture of cell soma distribution and non-overlap of marker expression as in comparable co-stains with parvalbumin instead of Zebrin II (**Figures 5C,C**’). Thus, these immunostains reveal that *shh-*GFP expressing zebrafish cerebellar cells are not calretinin-positive cells and confirm that they are not Purkinje cells because of a lack of parvalbumin expression.

**FIGURE 6:**
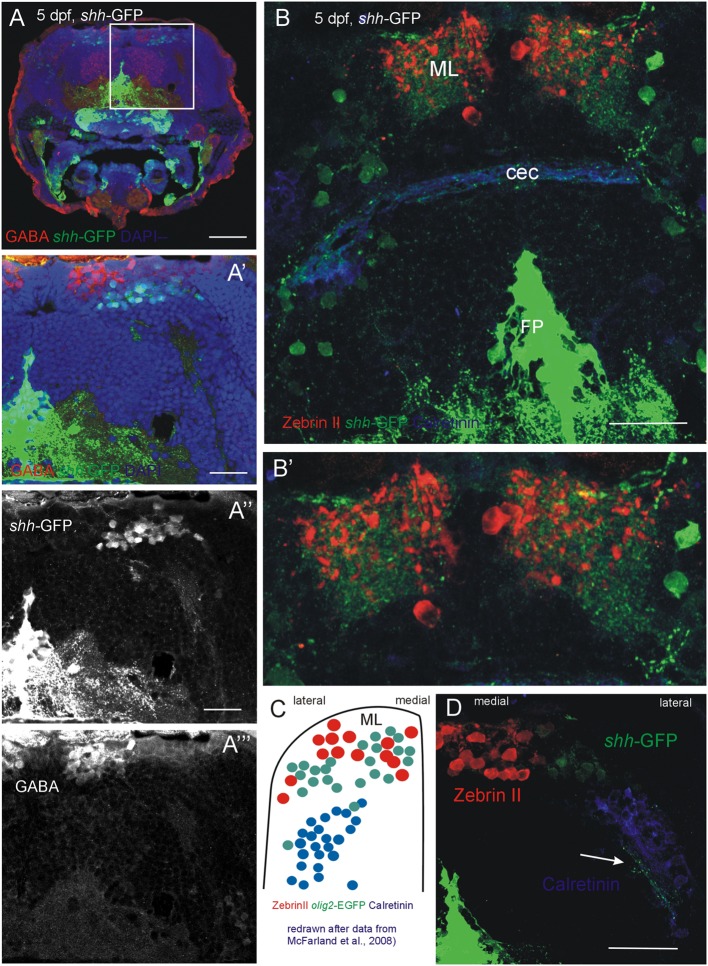
**Confocal photomicrographs (optical sections) of transverse sections of 5 dpf larval *shh-*GFP zebrafish cerebellum immunostained for GABA **(A–A”’)** or Zebrin II and Calretinin **(B,B’,D)**. (C)** Shows a drawing of data published by McFarland et al. (2008) to show correspondences of cerebellar cell populations shown in the present paper. Note stained axons of *shh*-GFP cells in (**D**; arrow). See text for details. Abbreviations: cec, cerebellar commissure; FP, floor plate; ML, molecular layer. Scale bars in **(A)**: 100 μm, in **(B,D)**: 50 μm, in **(A’)**: 20 μm.

In order to exclude the possibility that the *shh*-GFP cells are GABAergic cells other than Purkinje cells, such as stellate or Golgi cells, which might be present at this developmental time, an anti-GABA immunostain was performed in transgenic *shh*-GFP zebrafish larvae (**Figure 6**). These data show that the *shh*-GFP expressing cells are never co-localized with GABA positive cells, but form a completely separate population somewhat lateroventrally from the GABA immunopositive cells (**Figures 6A–A**”’). This eliminates the possibility that stellate or basket cells are expressing *shh*-GFP. As expected, GABA positive cells were more numerous than the Zebrin II or Parvalbumin populations because the latter only represent Purkinje cells, whereas the GABA positive cells also include cerebellar inhibitory interneurons.

Of particular interest is that the *shh-*GFP expressing cells lie in a position where *olig2* expressing eurydendroid cells had been reported previously (McFarland et al., 2008; **Figure 6C**). These *olig 2* expressing cells were found dorsomedially to the above mentioned calretinin positive cells in a position, which we describe here for cerebellar *shh-*GFP cells (**Figure 6D**). Furthermore, these cerebellar *shh-*GFP cells are not labeled with all other markers used here (Zebrin II, calretinin, Parvalbumin, GABA, as demonstrated above with confocal microscopy), and also not with PH3 (see next section). Moreover, the *shh*-GFP cell bodies extend a dendritic tree into the molecular layer (just as Purkinje cells do) which can be nicely seen in **Figures 6B,B**’ and which is expected of eurydendroid cells as well as of Purkinje cells in teleosts (see Section “Introduction”). This renders the *Ptf1a/olig2* positive zebrafish cerebellar eurydendroid cells as the only candidates for the *shh*-GFP expressing cells and they may thus perform a signaling function at the pial periphery where the proliferative EGL cells lie (see Section “Discussion”).

**FIGURE 7:**
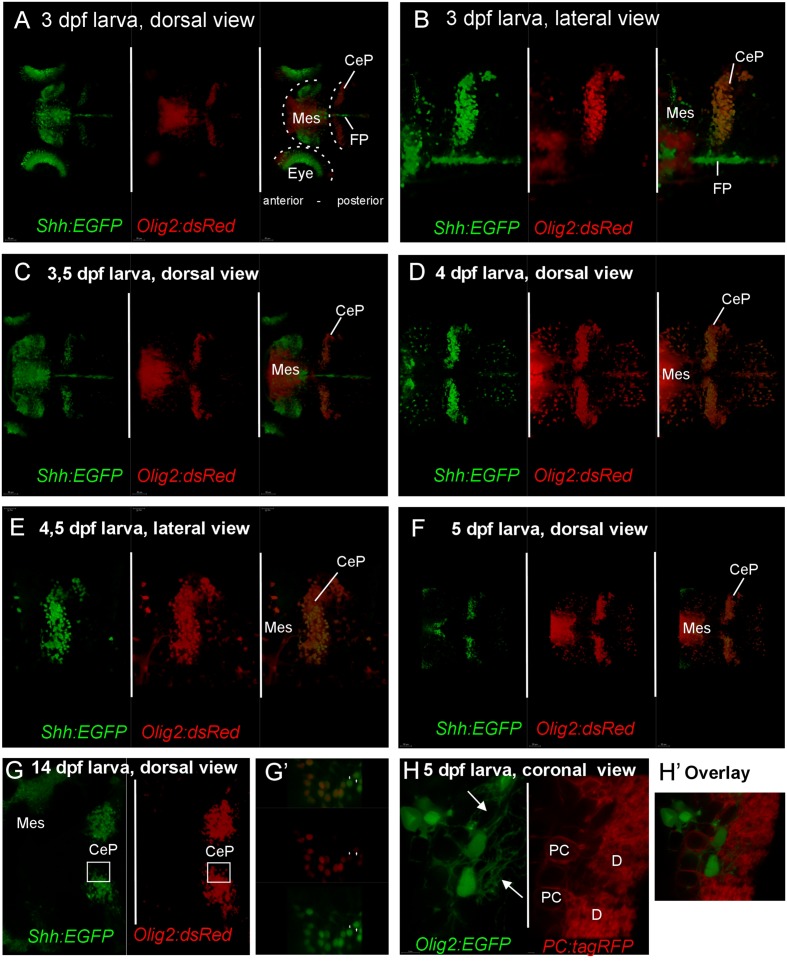
**Confocal live imaging of double-transgenic zebrafish larvae, i.e., Tg(*olig2:dsred2*)vu19/Tg(*2.4shha-ABC-GFP*)sb15 specimen, shows temporal development of *shh*-GFP and *olig2*-dsRed marked cells from 3 to 14 dpf **(A–G)**. (A–F)** Each panel shows two single label and one merged picture, **(G)** shows two single label pictures, with **G’** showing an enlarged overlay (optical section). Note that double labeled cells are restricted to the cerebellar plate and are absent from the midbrain. **(H)** is taken from a double-transgenic Tg(*olig2:EGFP*)/Tg(*PC:FyntagRFP-T*) larval zebrafish specimen at 5 dpf and shows a lateral region in the cerebellar plate. Note that *olig2*-*EGFP* expressing eurydendroid cells (green) lie close to *PC-tagRFP* positive Purkinje cells (red), but are never co-labeled. Note that green eurydendroid cells show dendrites extending into the molecular layer (arrows). **(H’)** shows an overlay. CeP, cerebellar plate; D, dendritic tree; FP, floor plate; Mes, mesencephalon (midbrain); PC, Purkinje cell body.

Finally, mating of *shh*-GFP fish with *olig2*-dsRed fish produced progeny that is double transgenically labeled for *shh* and *olig2* expressing cells and confocal live imaging was performed on such larvae between 3 and 5 dpf (**Figures 7A–F**). These experiments confirmed the conclusion of the detailed cellular marker analysis above and demonstrated that the *shh*-GFP cerebellar plate cells – in contrast to the midbrain cells – are always double-labeled for the *olig2*-dsRed as well, as exemplified in an optical section overlay from a fixed cerebellum of zebrafish as old as 14 dpf (**Figures 7G,G**’). Furthermore, larval (5 dpf) progeny of *olig2*-EGFP fish mated to *PC*-tagRFP fish demonstrates that *olig2* positive eurydendroid cells never co-localize with carbonic anhydrase 8 expressing Purkinje cells (**Figures 7H,H**’; see explanation of transgene in Section “Materials and Methods,” and Bae et al., 2009). Furthermore, the eurydendroid cell dendritic tree, nicely visualized with EGFP, is seen to extend between Purkinje cell bodies and to enter the cerebellar molecular layer (arrows in **Figure 7H**). Altogether, this is definite proof that the *shh*-GFP cells correspond to the *olig2* positive cerebellar eurydendroid cells.

### Presence of Mitotic Cells in External Granular Layer and Relationship to *shh-*GFP Cells

**FIGURE 8:**
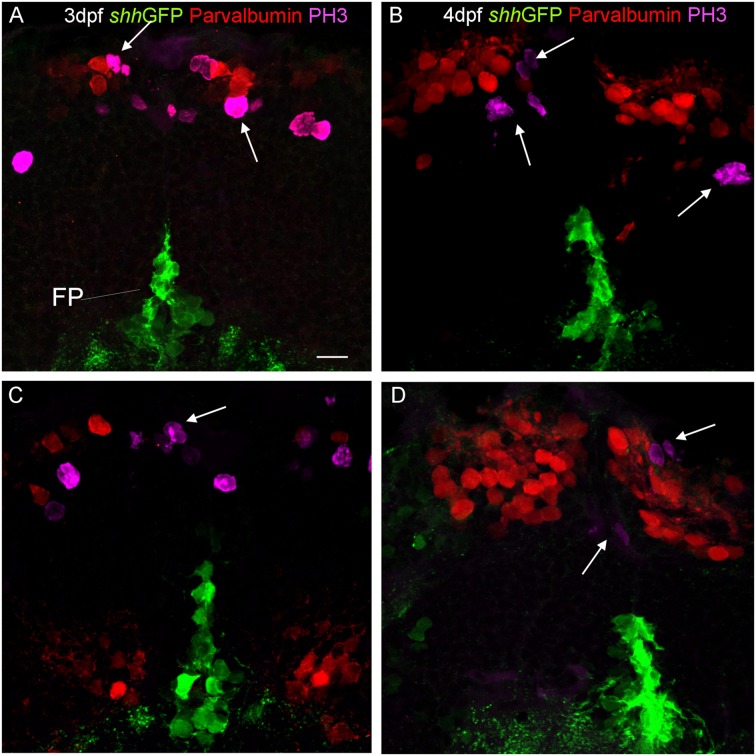
**Confocal photomicrographs (optical sections) of transverse sections of *shh-*GFP zebrafish cerebellum at 3 dpf **(A,C)** and 4 dpf **(B,D)** immunostained for parvalbumin and PH3.** Arrows point out mitotic PH3 positive cells which are neither GFP nor parvalbumin positive. Scale bar in **(A)**: 10 μm (applies to all panels).

**FIGURE 9:**
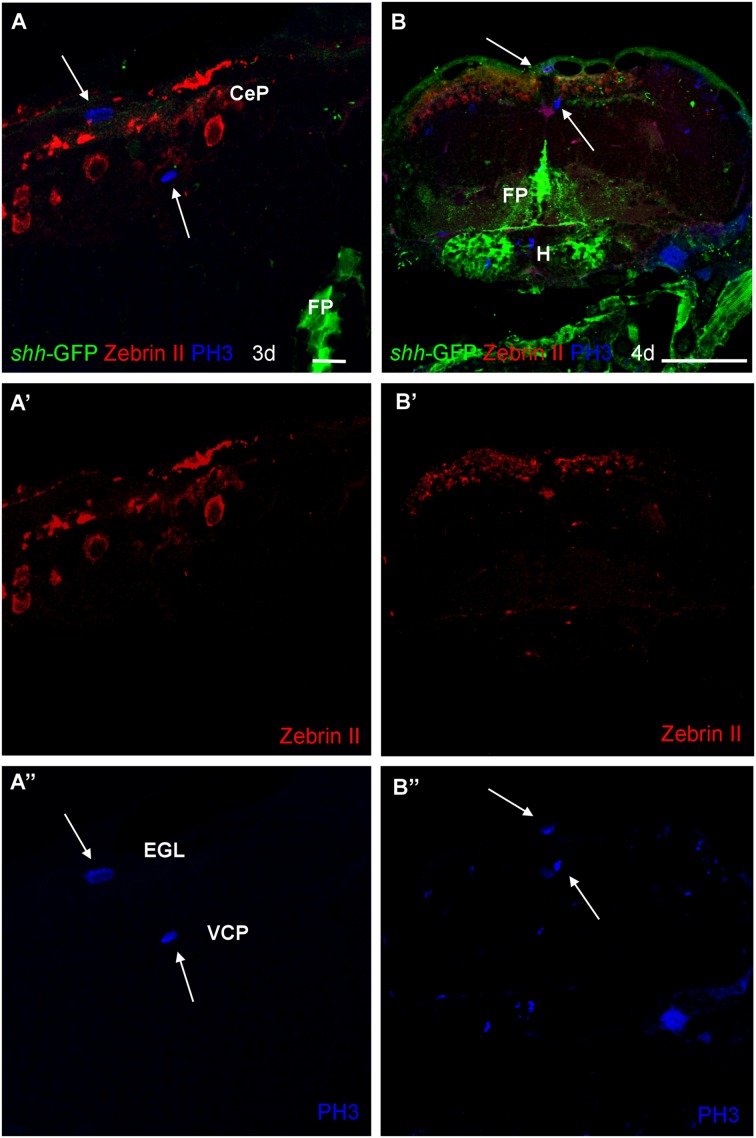
**Photomicrographs (optical sections) of transverse sections of *shh-*GFP zebrafish cerebellum at 3 and 4 dpf immunostained for Zebrin II and PH3.** Arrows point to mitotic PH3 positive cells which are never Zebrin II positive and lay basal or superficial to them. Scale bar in **(A)**: 10 μm (also applies to **A’**, **A”**) and in **(B)**: 100 μm (also applies to **B’**, **B”**). CeP, cerebellar plate; EGL, external granular layer; FP, floor plate; H, hypothalamus; VCP, ventral (ventricular) cerebellar proliferation zone.

Finally, in order to see whether or not the *shh-*GFP expressing cells are mitotic cells, we used antibodies against phospho-histone H3 (PH3), a most specific mitotic cell marker, in combination with either parvalbumin (**Figure 8**) or Zebrin II (**Figure 9**) in 3 and 4 dpf transgenic *shh*-GFP zebrafish larvae. In both assays, proliferative (mitotic) cells were seen in the ventral proliferative zone as well as toward the pial surface of the cerebellar plate in a position of an EGL, sandwiching the parvalbumin or Zebrin II positive Purkinje cells. The latter themselves were never PH3 positive (arrows in **Figures 8** and **9**). Also, PH3 was never co-localized in cells with the *shh*-GFP signal. The existence of the two proliferative zones had been evidenced previously with other markers (see Section “Discussion”), but the present combinatorial approach using PH3 is the most rigorous demonstration that both proliferation zones do exist in the larval zebrafish cerebellum. Because an EGL is characteristic of the cerebellum only, the optic tectum serves as an internal control here and, as expected, similar superficial proliferative cells are not seen in the optic tectum, but only basal, ventricular ones.

## Discussion

### The Transgenic *shh-*GFP Zebrafish Line Reveals Valuable Cytoarchitectonic Details Beyond Location of *shh* Expressing Cell Somata

The present analysis offers an overall description of *shh-*GFP expressing cell somata throughout the early larval zebrafish brain (2–5 dpf) and, thus, reveals conventionally established embryonic *shh* expressing structures in zebrafish (Krauss et al., 1993; Ekker et al., 1995; Holzschuh et al., 2003; Ertzer et al., 2007; Hagemann and Scholpp, 2012) and amniote vertebrates (Ericson et al., 1995; Shimamura et al., 1995) such as the retina, hypothalamus, zona limitans intrathalamica, and floor plate (see results, **Figures 1** and **2**, and Supplementary Figure 1). In addition, lesser known dorsal alar plate *shh* expressing cells later described in the embryonic mammalian isocortex, superior colliculus, and cerebellum (Dahmane and Ruiz i Altaba, 1999; Dahmane et al., 2001; Ruiz i Altaba et al., 2002) are partly visualized nicely in our combinatorial marker analysis. While no such cells are present in the zebrafish telencephalon, *shh-*GFP cells do show up in the optic tectum and cerebellum (see results). In contrast to a previous study which only reported *shh*-expressing cells in the zebrafish midbrain (Feijóo et al., 2011), we could clearly delineate such midbrain from cerebellar *shh* expressing cells by determination of cerebellar location using Zebrin II as an unambiguous marker of Purkinje cells (**Figure 2**).

Additionally, since GFP also enters axons and dendrites of *shh-*GFP cells, certain cytoarchitectonic details are revealed, some of which shall be discussed shortly. For example, axons of *shh-*GFP cells visualize nicely retinal projections to optic tectum and superficial pretectum (see Section “Results”). This further allows to differentiate between *shh-*GFP expressing cell somata in midbrain and cerebellum and confirmed additionally the presence of both populations. The GFP expressing axons seen in the lateral forebrain bundles likely represent ascending axons of GFP expressing posterior tubercular cells and not descending telencephalic axons, because no GFP expressing somatas were seen in the telencephalon. One of the main sources of telencephalic pallial input comes from the posterior tuberculum, likely including dopaminergic ones (Wullimann and Meyer, 1993; Rink and Wullimann, 2001, 2002, 2004; Northcutt, 2006).

Similar to the zebrafish *shh-*GFP expressing cells lateral to the floor plate, *shh* expressing cells have been described in rodents. Thus, Blaess et al. (2011) reported the presence of *shh* expressing cells increasingly more lateral to the floor plate between 8.5 and 12.5 embryonic days in the mouse midbrain. These cells are fated to become (dopaminergic) ventral tegmental area/substantia nigra compacta and nucleus ruber neurons, as well as astrocytes. It is therefore likely that also in the zebrafish, these lateral *shh-*GFP expressing cells develop into tegmental cell groups and astrocytes. However, in this location they will unlikely develop into dopaminergic neurons, since these are absent in the teleost midbrain (see Wullimann, 2014).

### Zebrin II Based Analysis of Zebrafish Purkinje Cell Distribution, Incl. Development, and Adult Configuration of the Cerebello-Octavolateralis Projection

Zebrin II positive Purkinje cells are present in the larval zebrafish cerebellar plate exhibiting nicely the extensively branching dendritic tree in the molecular layer emerging from the more basally located somata (**Figures 2–6**). There are no Zebrin II positive cells in the still small larval valvula cerebelli. The adult zebrafish cerebellum exhibits a monolayer of Zebrin II positive cell somata around the edge of the granular masses of the adult zebrafish corpus cerebelli (**Figure 4D**) with dendritic trees ascending into the molecular layer. Zebrin II positive Purkinje cells have also been reported in the adult zebrafish valvula cerebelli, in particular in the lateral division (Lannoo et al., 1991b). In the vestibulolateral lobe, an aggregate of Zebrin II positive cells is seen at the meeting point of corpus, eminentia granularis and caudal cerebellar lobe (**Figure 4D**). Some Zebrin II positive cells furthermore extend ventrally along the medial border of the eminentia granularis (arrowhead in **Figure 4E**’). A few cell bodies are also seen dorsal to the very caudal granular masses of the caudal lobe (**Figure 4F**).

Using Zebrin II immunohistochemistry, direct Purkinje cell projections to primary vestibular and mechanosensory lateral line areas (i.e., descending octaval nucleus and medial octavolateralis nucleus) have previously been described in two species of larval and adult S-American knifefishes and a catfish (Brochu et al., 1990; Lannoo et al., 1991a,b). However, beyond the presence of a larval tract (Lannoo et al., 1991a,b), its vestibular projection target and exact time point of origin (i.e., 5 dpf) have only recently been established in the zebrafish (Bae et al., 2009; Matsui et al., 2014; Tacheuki et al., 2015). We confirm here with Zebrin II immunohistochemistry the earliest occurrence of the cerebello-octavolateralis tract in the zebrafish larvae at 5 dpf, the position of larval Purkinje cells from which the tract originates in the caudal cerebellar plate and the exact course of these axons and their termination fields in the larval zebrafish vestibular brainstem area. What is more, we show that the adult location of the Purkinje cells, from which the cerebello-octavolateralis tract arises in the mature zebrafish, is in the vestibulolateral lobe (see Section “Results” and **Figure 4**) in a position where corpus cerebelli, eminentia granularis, and cerebellar caudal lobe meet. This confirms the identification of the vestibulolateral lobe as the homolog of the mammalian flocculo-nodular lobe, which also contains Purkinje cells projecting to vestibular nuclei (Walberg and Dietrichs, 1988; Nagao et al., 1997; Balaban et al., 2000; Barmack, 2003). Neurophysiological and neuroanatomical work in the goldfish and zebrafish has shown a function of vestibulolateral lobe circuits involving Purkinje cells and descending octaval nucleus in oculomotor performance (Pastor et al., 1994; Straka et al., 2006; Matsui et al., 2014). Additionally, the teleostean cerebello-octavolateralis tract also has terminations in the primary sensory lateral line nucleus, the medial octavolateralis nucleus (**Figures 4E–E**”), and the functional role of this projection has yet to be defined.

### Combinatorial Cellular Marker Analysis on the *shh-*GFP Background Reveals Identity of *shh* Expressing Alar Plate Cerebellar Cells

As reported above, we establish the existence of *shh*-GFP cells in the developing larval zebrafish cerebellar plate using Zebrin II, an ubiquitous Purkinje cell marker in teleosts, which allowed to differentiate midbrain from cerebellar *shh*-GFP cells (**Figures 2** and **3**). Furthermore, confocal microscopical analysis was used to delineate reliably cellular localization in this and all other multiple immunostains. This work demonstrated that the *shh*-GFP cells never co-localize with Zebrin II (**Figure 3**) and are therefore not Purkinje cells. Therefore, we started the search for the cellular identity of the *shh*-GFP cells. The calcium-binding proteins calretinin and parvalbumin characterize a rhombic lip derived migrating cell population (incl. mostly secondary gustatory nucleus cells) and Purkinje cells, respectively, in the zebrafish cerebellum (Bae et al., 2009; Volkmann et al., 2010). Double-immunostains in transgenic *shh*-GFP larvae for calretinin/parvalbumin or calretinin/Zebrin II allow us to conclude that *shh*-GFP cells are never co-labeled with any of those markers. They are therefore also in this assay confirmed neither to represent (parvalbumin-positive) Purkinje cells, nor to correspond to calretinin-positive eurydendroid cells. As expected, these immunostainings also show that parvalbumin and calretinin do not co-localize in cerebellar cells, confirming that different cerebellar cell types are visualized. Since additional inhibitory GABAergic cells can be expected to develop at these larval stages, anti-GABA immunostains were done in *shh*-GFP larvae. This showed a complete disjunct distribution with GABA-positive cells located dorsomedially to the ventrolateral *shh*-GFP cells, ruling out that *shh*-GFP cells represent inhibitory cerebellar cells (i.e., stellate cells, Golgi cells). In order to also exclude that *shh*-GFP cells are mitotic cells, parvalbumin-PH3 (**Figure 8**) and Zebrin II-PH3 immunostains (**Figure 9**) were performed in transgenic larvae. This shows that Purkinje cells (parvalbumin or Zebrin II positive ones) never co-localize with mitotic PH3 cells or with *shh*-GFP cells. The mitotic PH3 cells occur in the basal ventral cerebellar proliferation zone or in the cerebellar periphery, arguably in a position of an EGL. These two layers of PH3 cells “sandwich” differentiated, postmitotic cells.

**FIGURE 10:**
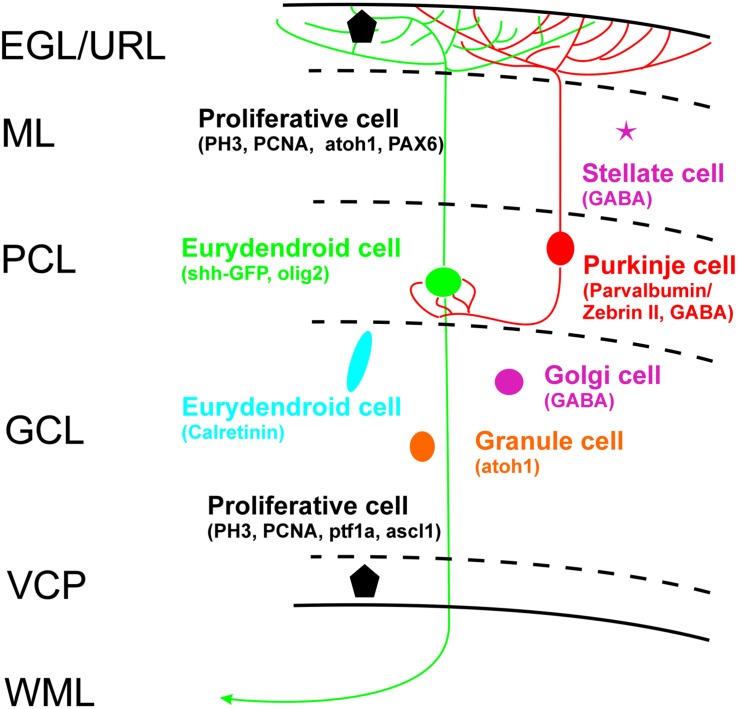
**Schema of zebrafish larval cerebellar plate organization summarizes cell types with characteristic cellular markers shown in the present study, with some additional data from Wullimann and Knipp (2000: PCNA), Wullimann and Rink (2001: PAX6), Mueller and Wullimann (2002: BrDU), Wullimann and Mueller (2002: ascl1a), Mueller et al. (2006: GABA), Kani et al. (2010: ptf1a/atoh1a-c), Wullimann et al. (2011: atoh1a-c).** EGL, external granular layer; GCL, granule cell layer; ML, molecular layer; PCL Purkinje cell layer; VCP, ventral (ventricular) cerebellar proliferation zone; WML, white matter layer.

Thus, if all the discussed cerebellar cells types can be ruled out as *shh*-GFP cells what is the identity of the latter? One remaining population are the eurydendroid cells which express the transcription factor *olig2* and which were demonstrated in a transgenic GFP-line to lie in a position ventrolateral to Zebrin II-positive cells within the zebrafish cerebellar plate, but dorsal to the calretinin-positive cell population that lies even more ventrolaterally (see **Figure 6C** after data from McFarland et al., 2008). The position of their olig2 positive cells corresponds closely to our *shh*-GFP population (see **Figure 6D** in comparison). What is more, our *shh*-GFP cells have an extensive dendritic tree into the molecular layer where it interdigitates with a similar dendritic tree formed by Purkinje cells (compare the red and green cell bodies and their fine arborizations in **Figures 6B,B**’). This is exactly what one would except of the morphology of eurydendroid and Purkinje cells in teleosts. Finally, our own live imaging experiments using double transgenic larval *shh*-GFP/*Olig2*-dsRed fish confirm this analysis because these specimens showed double labeled cells in the cerebellar plate, but not in the mesencephalon (**Figure 7**). Furthermore, in *Olig2*-EGFP/*PC*-tagRFP fish, the eurydendroid cell dendritic tree is clearly seen to extend beyond Purkinje cells into the cerebellar molecular layer. The various cellular markers investigated here, including some known from the literature are summarized in a sketch of the larval zebrafish cerebellar plate in **Figure 10**.

### Development of Upper Rhombic Lip Follows Largely Similar Ontogenetic Pattern As Seen in Amniotes Including the Presence of an External Granular Layer and Maybe Transit Amplification

**FIGURE 11:**
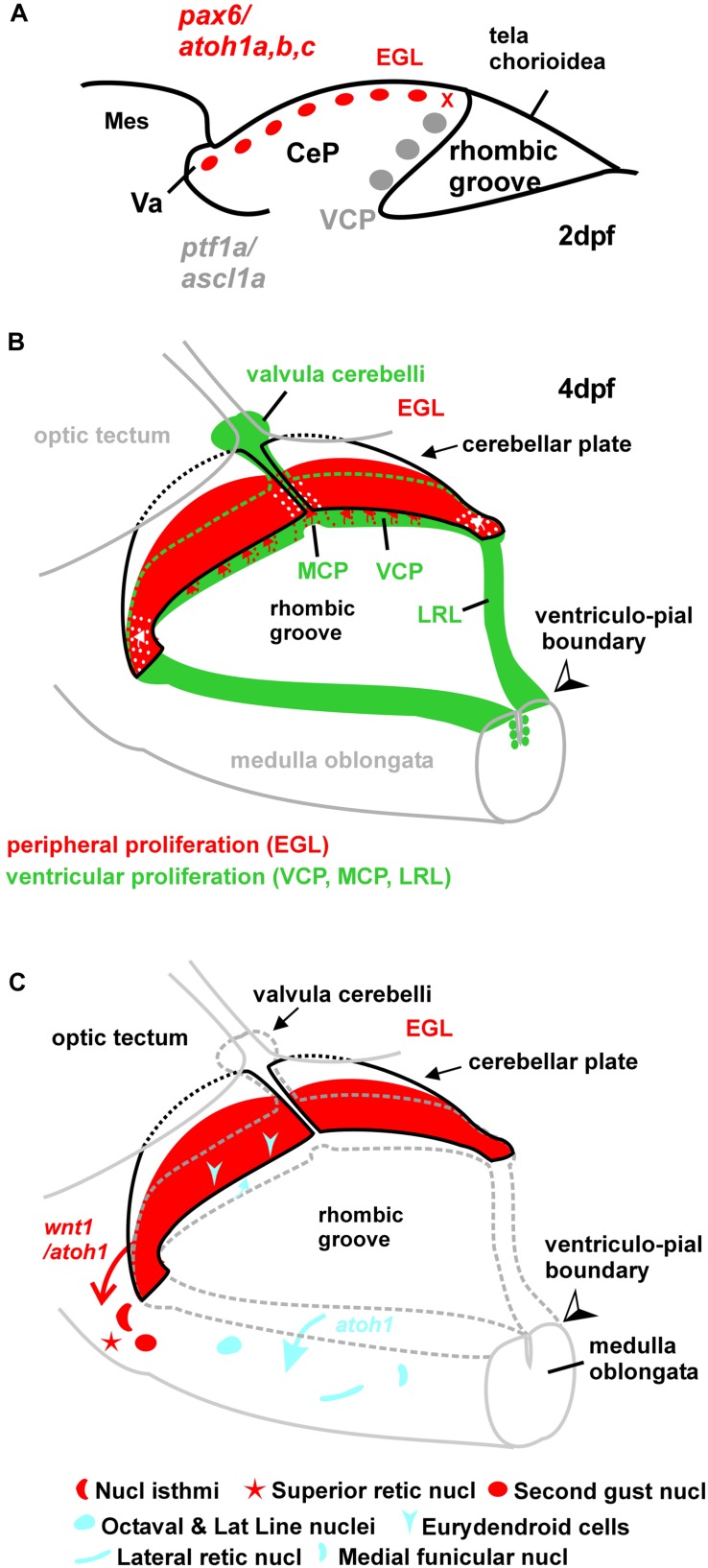
**Schematic drawings of zebrafish rhombic lip and cerebellar plate development. (A)** Sagittal section showing early larval zebrafish cerebellum (redrawn after Kani et al., 2010). The x indicates site of ventricular upper rhombic lip origin of subpial *atoh1* positive cells (see Wullimann et al., 2011). **(B)** Larval zebrafish rhombic lip (incl. cerebellar) proliferation zones. **(C)** Migration dynamics involving rhombic lip and external granular layer. The *wnt1* derived cholinergic nuclei in the isthmic region were demonstrated in zebrafish larvae (Volkmann et al., 2010). The *atoh1a* derived structures were observed in adult zebrafish transgenic *atoh1a*-GFP line (Wullimann et al., 2011). Note that the eurydendroid cells represent only the small *atoh1a* derived fraction. See text for details. CeP, cerebellar plate; EGL, external granular (or germinative) layer; LRL, lower rhombic lip; MCP, medial cerebellar proliferation zone; Mes, mesencephalon; MO, medulla oblongata; VCP, ventral cerebellar proliferation zone (ventricular germinal matrix); Va, valvula cerebelli.

On a broader scope, rhombic lip development in the zebrafish generally reveals to be highly comparable to the amniote condition outlined in the Section “Introduction.” We have reviewed these developmental similarities in detail previously (Wullimann et al., 2011). Briefly, there is a proliferative lower rhombic lip along the lateral edges of the rhombic groove as well as an anterior transversely oriented proliferative upper rhombic lip in the larval zebrafish brain (**Figure 11B**). While the neurogenic transcription factors *atoh1a* and *ptf1a* are segregated into adjacent dorsal and ventral domains, respectively, in the lower rhombic lip (Köster and Babaryka, personal observation), these two expression domains in the upper rhombic lip separate into a ventral *ptf1a* expressing one, seemingly associated with the ventral (ventricular) cerebellar proliferation zone (VCP), whereas a dorsal subpial *atoh1a* (and later *atoh1c*) expression domain is seen in a dorsal subpial location (**Figure 11A**). In both basal (VCP) and subpial (that is, an EGL) locations, proliferative cells are found with various assays, such as BrdU incorporation and PCNA or PH3 immunoassays (Mueller and Wullimann, 2002, 2003, 2005; Wullimann and Mueller, 2002; Volkmann et al., 2010; Wullimann et al., 2011; present contribution). Proliferation and neurogenesis in a subpial position is highly unusual to occur in the vertebrate neuroepithelium, but it is characteristic for the embryonic cerebellum in amniotes (Hatten, 1990; Hatten and Heintz, 1995). Thus, the mere existence of proliferative cells in the periphery of the zebrafish cerebellar plate is indicative of an EGL. Moreover, support comes from respective gene expression (*pax6*, *atoh1* genes subpially versus *ascl1a* and *ptf1a* ventrally; **Figure 11A**; after Kani et al., 2010). Since teleosts underwent an additional whole genome duplication in comparison to other vertebrates, often more than two paralogs are present, as it the case for *atoh1* (i.e., *atoh1a, b, c*) which are expressed in a temporal sequence. While *atoh1a/b* are expressed early, *atoh1c* continues to be expressed in a subpial cerebellar plate position at least up to 9 dpf (Wullimann et al., 2011) and PH3 positive cells are still seen there at this age, indicating that *atoh1c* progenitors are still produced. Opposed migratory paths of dorsally migrating *ptf1a* and ventrally migrating *atoh1a* derived cells and their fate as inhibitory (e.g., Purkinje) versus excitatory (granule) cells have been described (Kani et al., 2010). In line with this observation, differentiated (for example GABAergic) cells were described between the two proliferation zones of the zebrafish cerebellar plate (Mueller and Wullimann, 2002, 2005). However, unlike the *atoh1* dependent excitatory deep cerebellar nuclear cells in amniotes, zebrafish eurydendroid cells are in their overwhelming majority derived from *ptf1a* expressing progenitors (Kani et al., 2010). Moreover, an early ventral migration of *wnt1/atoh1* dependent upper rhombic lip cells into the rostral medulla oblongata was documented to occur in similar fashion as in amniotes (Volkmann et al., 2010). Such cells were shown to be cholinergic and to have an early differential profile of connections with diencephalon and mesencephalon known in adult teleosts for the nucleus isthmi, secondary gustatory and superior reticular nucleus (**Figure 11C**) and, thus, represent the homologs of the parabigeminal, parabrachial and laterodorsal tegmental nuclei in mammals sharing an identical developmental upper rhombic lip origin. Furthermore, adult expression studies using an *atoh1a*-GFP transgenic line showed GFP expression in various precerebellar nuclei (lateral reticular, medial funicular nuclei, rostral dorsal horn), the primary sensory lateral line medial octavolateralis nucleus and octaval primary sensory nuclei as well as in a few eurydendroid cells consistent with an origin of these nuclei/cells from *atoh1a* expressing upper rhombic lip cells (Kani et al., 2010; Wullimann et al., 2011; 11C). Overall these data document an impressively close correspondence of temporal events in rhombic lip development between amniotes and teleosts, including the occurrence of a subpial proliferative, *atoh1* expressing domain, that is, an EGL.

In the present paper, we show that postmitotic, differentiated eurydendroid cells express the signaling molecule Shh analogous to what embryonic Purkinje cells do in mammals. The different developmental genetics of eurydendroid cells (mostly p*tf1a* dependent, instead of *atoh1a* dependent like amniote deep cerebellar nuclei neurons) may be causal to their altered migration behavior and the resulting location of these cells within the cerebellar plate (and not at the base of the cerebellum as in amniotes). This location is accompanied by the development of a cellular morphology different from mammalian deep cerebellar nuclei neurons, that is, eurydendroid cells establish an extensive dendritic tree into the molecular layer.

Altogether, this represents the precondition for such teleostean eurydendroid cells to potentially function in Shh signaling to EGL cells in transit amplification which is suggested by their being those cells with *shh-*GFP expression as shown in this paper. Interestingly, in the early embryo the number of these cerebellar *olig2* dependent eurydendroid cells is apparently regulated by the well known antagonistic activity of *shh* and *wnt1* across the dorsoventral neural tube axis (McFarland et al., 2008) by way of their floor plate and roof plate expression. Based on our findings, the actual occurrence of transit amplification in the teleostean cerebellar plate has to be investigated more rigorously now with knock-in and knock-out experiments. The secreted Shh protein binds to its receptor Patched, a 12-pass transmembrane receptor which acts by suppressing the activity of Smoothened, a G-protein coupled receptor. Activation of Smoothened results from some conformational change allowing activation of intracellular targets associated with the cytoskeleton. These include three zinc-finger transcription factors coding for Gli proteins (Gli1, Gli2, and Gli3) that in consequence regulate the transcription of target genes in hedgehog signaling in a combinatorial fashion (Traiffort et al., 1998). These and other players in the hedgehog pathway need further investigation in the zebrafish cerebellum.

## Conclusion

We have been asking the larval zebrafish over more than a decade whether there is a cerebellar external granular layer using various assays for proliferation, gene expression, migration, and differentiation. The answer has recurrently and consistently been that there are proliferating cells at the dorsal pial periphery of the larval cerebellar plate in addition to proliferating cells in the ventral cerebellar ventricular zone and that differentiating and migrating cerebellar cells are sandwiched between them (Mueller and Wullimann, 2002, 2003, 2005; Wullimann and Mueller, 2002; Volkmann et al., 2010; Wullimann et al., 2011). In the present contribution we can restate that the most rigorous assay for mitotic cells, PH3, gives us the same answer again for the zebrafish larval cerebellum. Moreover, the use of the *shh-*GFP transgenic line reveals not only the presence of *shh* expressing cells in the alar plate of mesencephalon (tectum) and cerebellum, as is the case in amniotes, but our analysis of critical cerebellar markers suggests furthermore that larval zebrafish eurydendroid cells represent this *shh-*GFP cerebellar cell type. Because they develop from p*tf1a* progenitors, do not migrate out of the cerebellum, and have a dendritic tree into the molecular layer similar to Purkinje cells, they qualify for a function in transit amplification as known for embryonic Purkinje cells in amniotes.

## Author Contributions

DB and MFW wrote the first draft, RWK, BG, and AD contributed to the final version of the article. The data were generated by DB/MFW except for those shown in **Figure 7** which were generated by RWK/AD. All authors listed, have made substantial, direct and intellectual contribution to the work, and approved it for publication.

## Conflict of Interest Statement

The authors declare that the research was conducted in the absence of any commercial or financial relationships that could be construed as a potential conflict of interest.
