# Supplementary material for: Eppur Si Muove: Evidence for an External Granular Layer and Possibly Transit Amplification in the Teleostean Cerebellum
Source: Front Neuroanat. 2016 May 2;10:49. doi: 10.3389/fnana.2016.00049 (PMC4852188; doi:10.3389/fnana.2016.00049)
Supplement: Supplementary file 1 [file Presentation_1.PDF]

Supplementary Materials:

Supplementary Fig. 1: Comparison of retina of zebrafish *shh*-GFP line with immunostained wildtype larval and adult zebrafish (confocal photomicrographs, except E). (A-D) Sections of retina in transgenic zebrafish at 2, 3, 4, and 5 dpf show GFP-positive amacrine and ganglion cells in addition to fiber net in internal plexiform layer. (D') Same section as (D) shows retinal layers. Note monolayer of photoreceptors and multiple ganglion cell layers. (E) Wildtype larval zebrafish retina stained immunohistochemically for GABA, visualizing amacrine (incl. some that are displaced into ganglion cell layer) and a few horizontal cells. (F-H) Wildtype adult zebrafish retina stained immunohistochemically for GABA and tyrosine hydroxylase (TH) visualizing amacrine cells. Magnification in (F) shows GABAergic amacrine cells in inner nuclear layer and displaced ones (green) next to ganglion cells (blue) and one dopaminergic amacrine (interplexiform) cell (red). Note the latter's dendritic punctate staining in the inner part of the outer plexiform layer. Magnification in (H) shows cell somata layers with DAPI. Note in inner nuclear layer the lighter stain of amacrine cells compared to bipolar cells and the elongated shape of horizontal cells. Counterstains: DAPI and NeuroTrace (NT). Asterisks: proliferative retinal edge. Scale bar in (A): 100µm (applies to panels B-E), in (G): 50µm. Abbreviations: AC amacrine cells (retina), dAC displaced amacrine cells, DA-AC dopaminergic amacrine (interplexiform) cells, GC ganglion cell, GCL ganglion cell layer, HC horizontal cells, INL inner nuclear layer, IPL inner plexiform layer, ONL outer nuclear layer, OPL outer plexiform layer, R/C rods/cones (inner & outer segments), RPE retinal pigment epithelium

Comparison with original transgenic *shh*-GFP line description for retina

The original description of the zebrafish *shh*-GFP line (Shkumatava et al., 2004; Stadler et al., 2004) focused on the role of Sonic Hedgehog in the differentiation of ganglion and amacrine cells in the developing retina. We observe identical developmental profiles of a first wave of *shh*-GFP expressing ganglion cells followed by a second wave of *shh*-GFP-expressing amacrine cells between 2 and 5 dpf (Suppl. Fig. 1). In the 2 dpf zebrafish, a limited number of positive ganglion cells is present in the ventral to central retina towards the vitreous body (Suppl. Fig. 1A). The proliferative peripheral edge of the retina (asterisks in Suppl. Fig. 1) is always free of a GFP signal at 2 dpf and older larvae. In the 3 to 5 dpf zebrafish retina, amacrine cells start to appear, as well as increasingly more ganglion cells become labelled, including the processes of both cell types in the internal plexiform layer (Suppl. Fig. 1B-D). Even earlier than these events, *shh* begins to be expressed in the retinal

pigment layer where it has a signaling role for photoreceptor differentiation (Stenkamp et al, 2000; Stenkamp and Frey, 2003). There is also some GFP signal in the larval photoreceptor layer. Larval retinal amacrine cells may be identified by comparing the GFP expressing cells in the internal plexiform layer to a larval wild type retina stained for GABA (Suppl. Fig. 1E; with nuclear counterstain DAPI and fluorescent Nissl stain NeuroTrace; see Mueller et al., 2006, for details). Such immunopreparations show a fraction of amacrine cells to be GABAergic (including displaced amacrine cells) as well as a minor number of horizontal cells at the inner side of the outer plexiform layer (Suppl. Fig. 1E). As expected, no GABA positive ganglion cells are seen. Also, non-GABAergic amacrine cells are evident (Suppl. Fig. 1E), likely dopaminergic (Jang et al., 2011) and cholinergic (Paulsen et al, 2010) ones.

Both types of GABAergic amacrine cells and the horizontal cells are present in the adult zebrafish retina (Suppl. Fig. 1F-H), next to very regularly spaced dopaminergic amacrine (interplexiform) cells, additionally visualized here using an antibody against tyrosine hydroxylase. These amacrines were never double-labeled for GABA and - similar to the situation in the mammalian retina (Contini and Raviola, 2002, Zhang et al., 2008) - nicely show dendritic staining in the inner part of the outer plexiform layer (Suppl. Fig. 1F; G), in addition to dendritic staining in the inner plexiform layer and around GABAergic amacrines. The multilayered larval ganglion cell layer (Suppl. Fig. 1E) thins out into an adult monolayer (Suppl. Fig. 1F-H) whereas the opposite is seen in the outer nuclear layer. The latter only consists of one larval photoreceptor somata layer in the central retina (Suppl. Fig. 1D, D', E; see also Schmitt and Dowling, 1999) which apparently increases subsequently in thickness and attains a pseudolayered structure in the adult zebrafish (Suppl. Fig. 1F-H), underlining the different dynamics of cell production in the ganglion and photoreceptor cell layers. Because rod nuclei in the larval zebrafish retina lie somewhat vitreal to cone nuclei (Schmitt and Dowling, 1999), the apparent two adult ONL layers thus likely represent rod nuclei (smaller, intense DAPI stain) and cone nuclei (larger, weaker DAPI stain). Adult zebrafish retinal cell types have been extensively studied by Connaughton and colleagues (e.g., 2001; 2004; 2011), Marc and Cameron (2001) and Yazulla and Studholme (2001).

Supplementary Fig. 2: Zebrafish (wild type, WT) brain section in-*situ* hybridized for *shha* at 5 dpf and analyed with confocal microscopy shows expected expression domains (right panels). Left panels: corresponding DAPI pictures for anatomical identification. Upper row: level of zona limitans intrathalamica (ZLI). Middle row: level of optic tectum (TeO) and cerebellar plate (CeP; boundary shown by red stippled line) shows *shha* expressing cells in floor plate

(FP) and cerebellar plate, as well as in pharynx. Bottom row: level of posterior cerebellar plate (boundary towards medulla oblongata, MO, indicated by red arrowheads in left panel), *shha* expressing cells in FP and cerebellar plate (encircled in red), as well as in pharynx.

Supplementary Fig. 3: Zebrafish transgenic *shh*-GFP line brain sections in-*situ* hybridized for *shha* at 5 dpf and analyed with confocal microscopy shows *shha* expression domains as seen in WT (compare to Supplementary Fig. 2) to largely overlap with GFP. Upper row shows overview of valvular cerebellar (Va) level, bottom row is an enlargement. Left panels: DAPI, middle panels: *shha*, right panels: *shhGFP*. Red arrowheads indicate border between optic tectum (TeO) and valvula (Va), red circle indicates region of interest. Medulla oblongata is separated from cerebellum by rhombencephalic ventricular space. Importantly, there are dorsal cerebellar cells double-labeled for the transgene (*shhGFP*) and the in-*situ* signal (*shha,* red arrows).

Supplementary References

Connaughton, V.P. (2001) Organization of ON- and OFF-pathways in the zebrafish retina: neurotransmitter localization, electrophysiological responses of bipolar cells, and patterns of axon terminal stratification. In: Kolb, H., Ripps, H., Wu, S. (Eds), Progress in Brain Research, Vol. 131: 161-176.

Connaughton, V.P. (2011) Bipolar cells in the zebrafish retina. Visual Neuroscience 28: 77-93.

Connaughton, V.P., Graham, D., Nelson, R. (2004) Identification and morphological classification of horizontal, bipolar, and amacrine cell within the zebrafish retina. J. Comp. Neurol. 477: 371-385.

Contini, M., and Raviola, E., (2003). GABAergic synypses made by a retinal dopaminergic neuron. PNAS 100: 1358-1363.

Jang, Y.-J., Yu, S.-H., Lee, E.-S., Jeon, C.-J. (2011). Two types of tyrosine-hydroxylase immunoreactive neurons in the zebrafish retina. Neuroscience Research 71: 124-133.

Marc, R.E., and Cameron, D. 82001) A molecular phenotype atlas of the zebrafish retina. J. of Neurocytology 30: 593-654.

Mueller, T., Vernier, P., and Wullimann, M.F. (2006). A phylotypic stage in vertebrate brain development: GABA cell patterns in zebrafish compared with mouse. J. Comp. Neurol. 494: 620–634

Paulsen, U.J., Tiedemann, K., Mack, A.F. (2010). Dendritic differentiation in the periphery of the growing zebrafish retina. Exp. Brain Res. 90: 514-520.

Schmitt, E.A., and Dowling, J.E. (1999). Early retinal development in the zebrafish, *Danio rerio*: light and electron microscopic analyses. J. Comp. Neurol. 404: 515-536.

Shkumatava, A., Fischer, S., Müller, F., Strahle, U., and Neumann, C.J. (2004). Sonic hedgehog, secreted by amacrine cells, acts as a short-range signal to direct differentiation and lamination in the zebrafish retina. Dev. 131: 3849-3858.

Stadler, J.A., Shkumatava, A., Neumann, C.J. (2004). The role of hedgehog signaling in thel development of the zebrafish visual system. Dev Neuroscie 26: 346-351 (DOI: 10.1159/000082276)

Stenkamp, D.L., and Frey, R.A. (2003). Extraretinal and retinal hedgehog signaling sequentially regulare retinal differentiation in zebrafish. Devel. Biol. 258: 349-363.

Stenkamp, D.L., Frey, R.A., Prabhudesai, S.N., Raymond, P.A. (2000) Function for *hedgehog* genes in zebrafish retinal development. Devel. Biol. 220: 238-252.

Yazulla, S., and Studholme, K.M. (2001) Neurochemical anatomy of the zebrafish retina as determined by immunocytochemistry. J. Neurocytol. 30: 551-592.

Zhang, D.-Q. Wong, K.Y., Sollars, P.J., Berson, D.M., Pickard, G.E., McMahon, D.G. (2008) Intraretinal signaling by ganglion cells photoreceptors to dopaminergic amacrine neurons. PNAS 105: 14181-14186
