# Supplementary figures and images for: Eppur Si Muove: Evidence for an External Granular Layer and Possibly Transit Amplification in the Teleostean Cerebellum
Source: Front Neuroanat. 2016 May 2;10:49. doi: 10.3389/fnana.2016.00049 (PMC4852188; doi:10.3389/fnana.2016.00049)

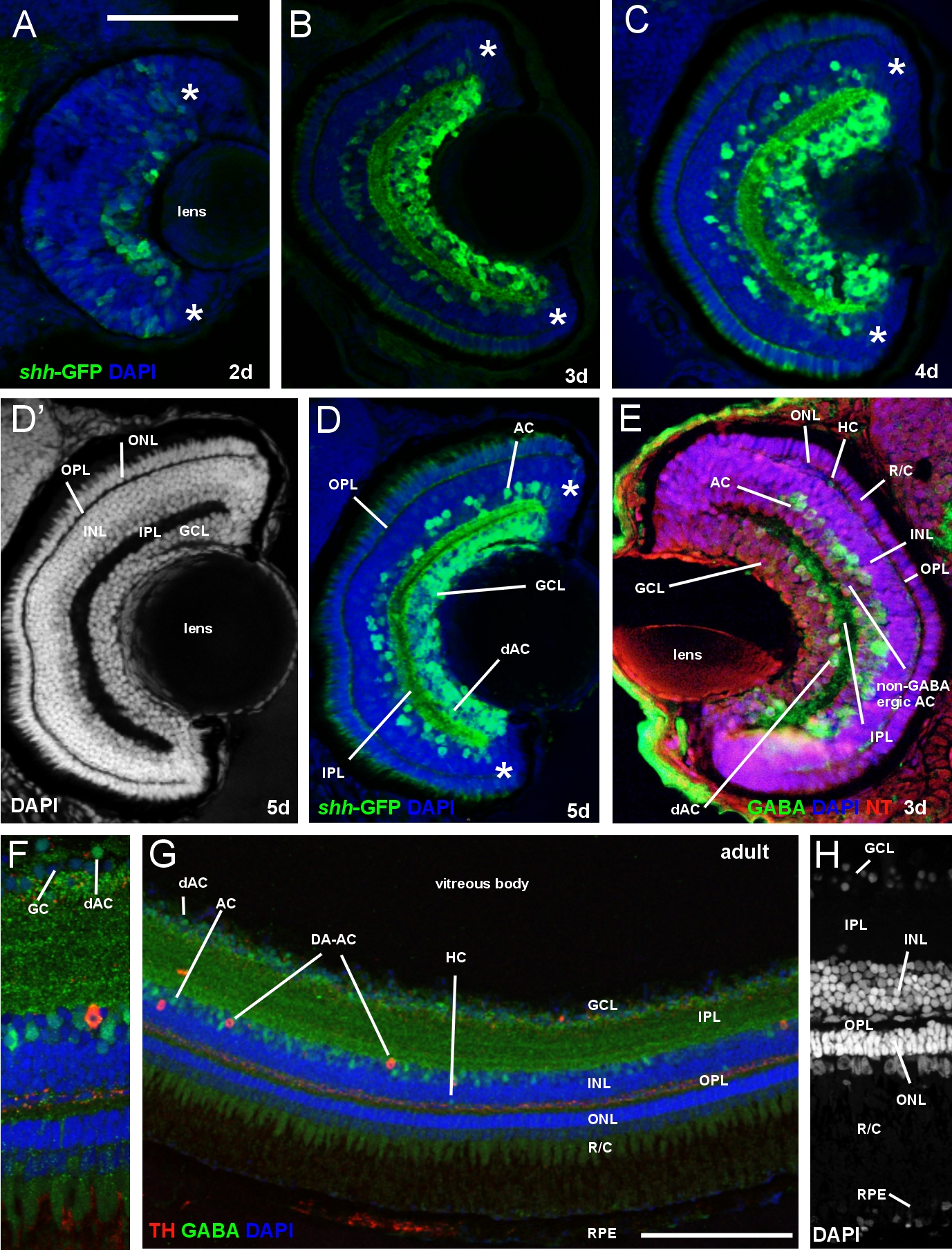

Supplement: Supplementary file 2 [file Image_1.JPEG]

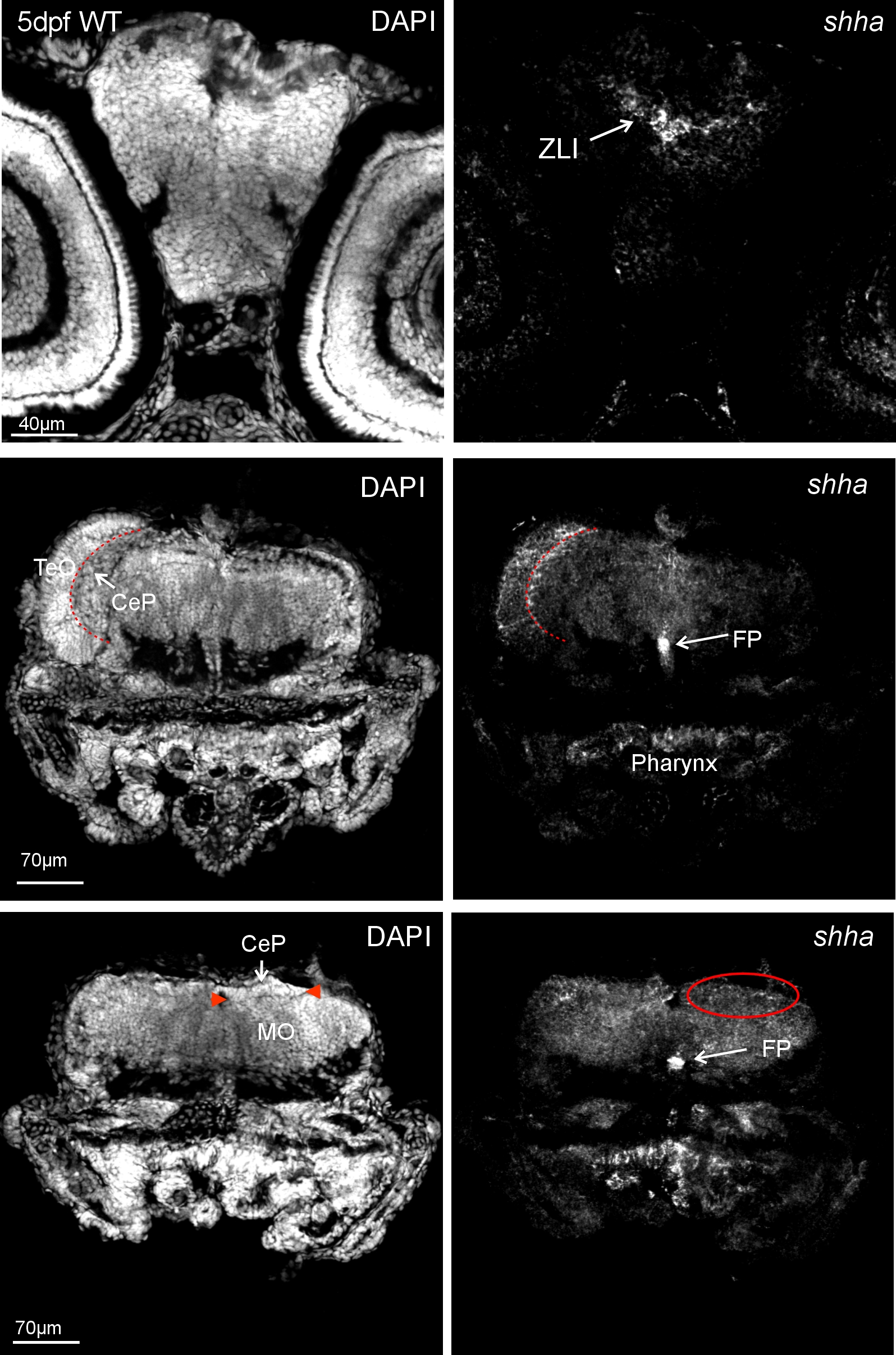

Supplement: Supplementary file 3 [file Image_2.JPEG]

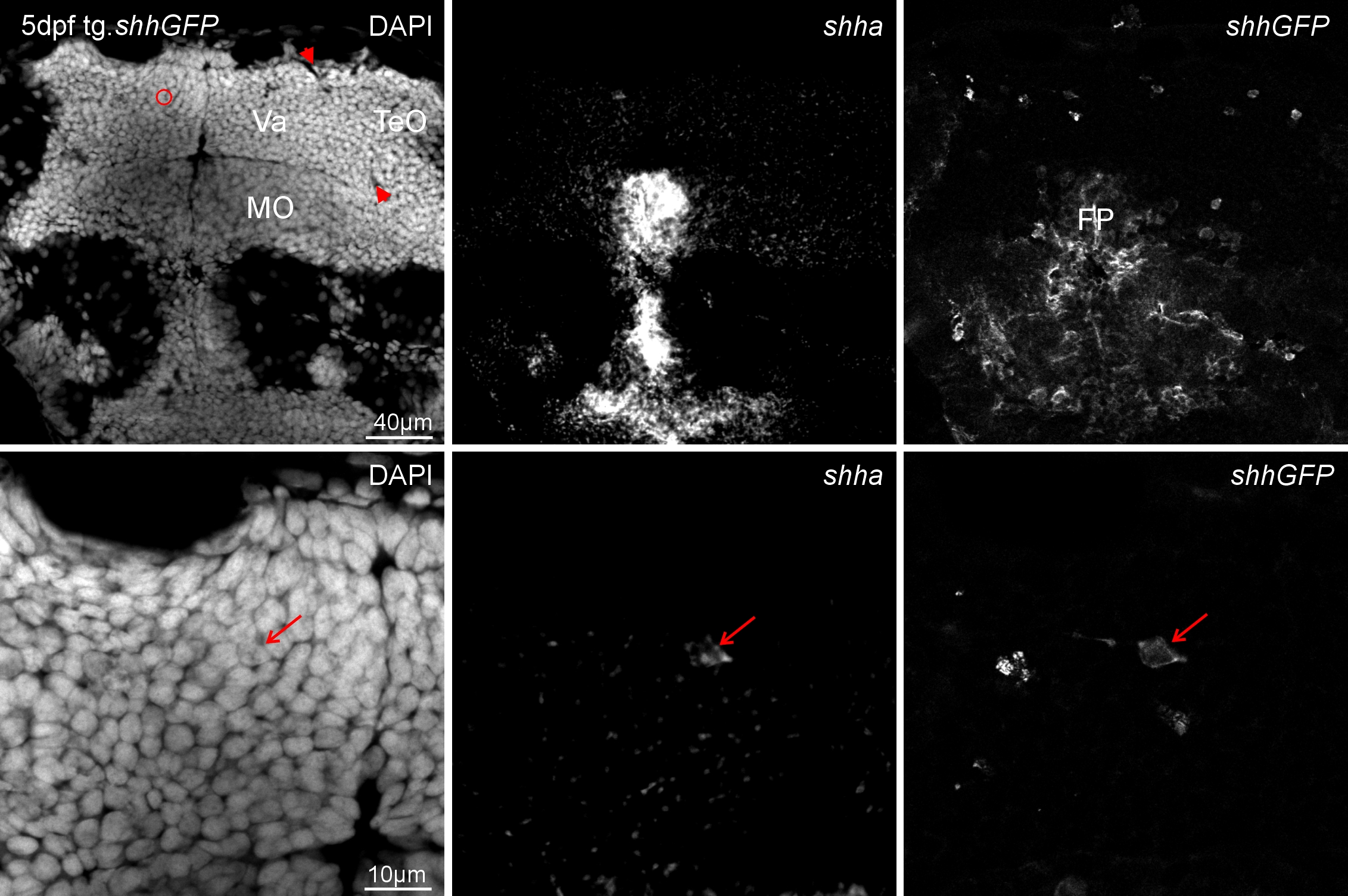

Supplement: Supplementary file 4 [file Image_3.JPEG]
